# Characterization of Anti-Inflammatory and Antioxidant Constituents from *Scutellaria baicalensis* Using LC-MS Coupled with a Bioassay Method

**DOI:** 10.3390/molecules25163617

**Published:** 2020-08-09

**Authors:** Yoo Kyong Han, Hyunwoo Kim, Hyeji Shin, Jiyeon Song, Mi Kyeong Lee, Byoungduck Park, Ki Yong Lee

**Affiliations:** 1 College of Pharmacy, Korea University, Sejong 30019, Korea; yookyong05@korea.ac.kr (Y.K.H.); hjshin90@korea.ac.kr (H.S.); sjy777777@naver.com (J.S.); 2 Center for Marine Biotechnology and Biomedicine, Scripps Institution of Oceanography, La Jolla, CA 92093, USA; hwkim8906@gmail.com; 3 College of Pharmacy, Chungbuk National University, Cheongju 28160, Korea; mklee@chungbuk.ac.kr; 4 College of Pharmacy, Keimyung University, Daegu 42601, Korea

**Keywords:** *Scutellaria baicalensis*, Labiatae, LC-MS coupled with bioassay, chemical profile, anti-inflammatory, antioxidant, molecular networking

## Abstract

An effective and previously demonstrated screening method for active constituents in natural products using LC-MS coupled with a bioassay was reported in our earlier studies. With this, the current investigation attempted to identify bioactive constituents of *Scutellaria baicalensis* through LC-MS coupled with a bioassay. Peaks at broadly 17–20 and 24–25 min on the MS chromatogram displayed an inhibitory effect on NO production in lipopolysaccharide-induced BV2 microglia cells. Similarly, peaks at roughly 17–19 and 22 min showed antioxidant activity with an 2,2′-azino-bis(3-ethylbenzothiazoline-6-sulfonic acid) (ABTS)/2,2-diphenyl-1- picrylhydrazyl (DPPH) assay. For confirmation of LC-MS coupled with a bioassay, nine compounds (**1**–**9**) were isolated from an MeOH extract of *S. baicalensis*. As we predicted, compounds **1**, **8**, and **9** significantly reduced lipopolysaccharide (LPS)-induced NO production in BV2 cells. Likewise, compounds **5**, **6**, and **8** exhibited free radical-scavenging activities with the ABTS/DPPH assay. In addition, the structural similarity of the main components was confirmed by analyzing the total extract and EtOAc fractions through molecular networking. Overall, the results suggest that the method comprised of LC-MS coupled with a bioassay can effectively predict active compounds without an isolation process, and the results of molecular networking predicted that other components around the active compound node may also be active.

## 1. Introduction

Natural products, which have a variety of chemical structures and are produced by diverse organisms (microorganisms, plants, animals, and humans), possess potential therapeutic properties and are fascinating drug leads [1]. The isolation of active components from natural products is normally achieved by bioassay-guided purification methodologies, but this often results in higher rates of unnecessary reisolation and dissipation of bioactive compounds from repeated processes [1,2].

Recently, liquid chromatography-mass spectrometry (LC-MS) or nuclear magnetic resonance (LC-NMR) methods were applied as dereplication tools to determine known compounds before beginning any isolation step [2]. Dereplication is an essential stage in the development of natural product drugs, and LC-MS can be used as the main technique for the overall profiling and dereplication of natural product extracts, which are usually complex mixtures [3,4]. An LC-MS coupled method is streamlined because it allows the annotation of new and known components while prioritizing them systematically so that they can be targeted and distinguished [5]. There is also a need for infrastructure that can split and aggregate data, even though LC-MS is well-suited to the massive processing of natural products [6]. MS/MS molecular networking was described as an effective tool in visualizing for dereplication [7]. Molecular networking is established through networking the chemical and structural similarity relationships between metabolites based on the similarity of their MS/MS fragments. Thus, metabolites with similar scaffolds can be grouped into clusters, confirming structural similarity [2,3,6]. This suggests the possibility of identification for unidentified nodes, if their spectra or the spectra of surrounding nodes are known by reference. Recently, molecular networking was introduced in drug development and metabolomics, particularly for natural products containing hundreds of components [8]. Molecular networking is mainly conjugated for prioritization specific structures that are expected to be active [2,3,4] and for annotation of bioactive extracts in natural products [5,9].

*Scutellaria baicalensis* Georgi is a perennial herb of the Labiatae native to East Asia and Russia [10]. The dried root of *S. baicalensis* (Scutellariae Radix) is used in traditional Chinese medicine for fever clearance and intoxication [11]. Moreover, recent studies demonstrated that *S. baicalensis* has pharmacological effects, such as antitumor [12], hepatoprotective [13], anti-inflammatory [14], and antioxidant activities [15]. The reported phytochemical constituents of *S. baicalensis* are mainly related to flavones, such as baicalin, wogonoside, chrysin, and their glycosides [16].

In a previous study, LC-MS was coupled with a cell-based method for dereplication, efficiently identifying NO production inhibitors associated with anti-inflammatory activity [17]. In this study, we attempted to find bioactive components from *S. baicalensis* using LC-MS coupled with a bioassay. In addition, the association between structural similarity and bioactivity to components of *S. baicalensis* was demonstrated through molecular networking and LC-MS coupled with a bioassay method.

## 2. Results and Discussion

### 2.1. LC-Quadrupole Time of Flight (QTOF) MS/MS Coupled with Bioassay

**Figure 1:**
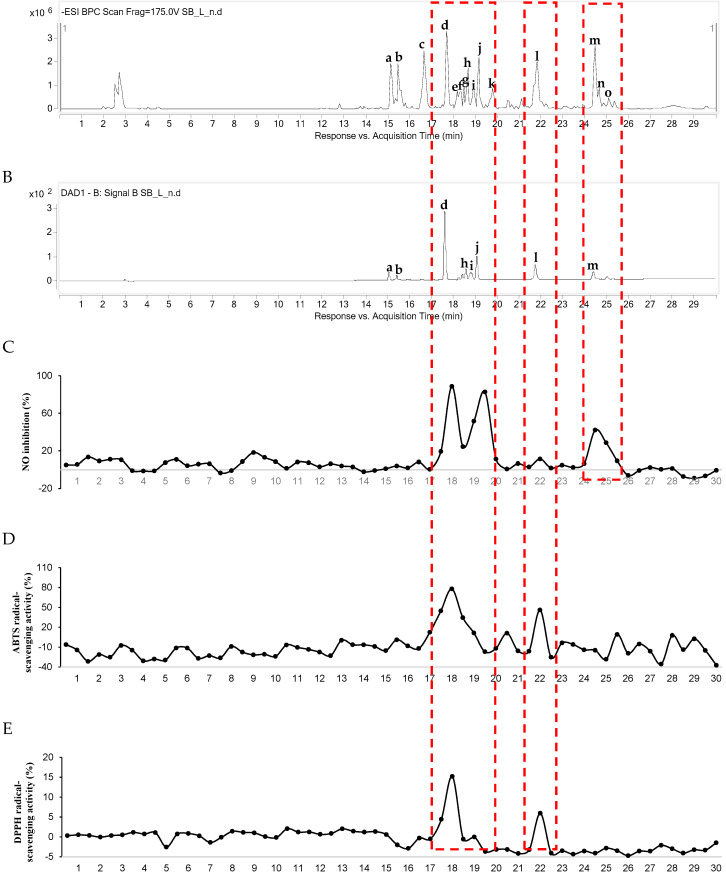
LC-QTOF MS/MS coupled with bioassay. (**A**) ESI-MS chromatogram (negative ionization mode); (**B**) UV chromatogram (254 nm); (**C**) NO production inhibitory activity of each 30 s intervals eluent; (**D**) ABTS free radical-scavenging activity of each 30 s intervals eluent; (**E**) DPPH free radical-scavenging activity of each 30 s intervals eluent of MeOH extract of *S. baicalensis.*

For LC-QTOF MS/MS coupled with bioassay, the first phase was to obtain the chemical profile of the MeOH extract of *S. baicalensis* (Figure 1A,B, Figure S1 and Table 1), and the second phase was to collect the eluent through the column for 30 s per well in a 96-well plate. The collected sample was used for LC-MS coupled with a bioassay for NO production along with an ABTS/DPPH-determined free radical-scavenging activity assay (Figure 1C–E). The results of the inhibition of NO production indicated that the constituents at 17–20 and 24–25 min on the MS chromatogram featured inhibitory activity. The results of the ABTS and DPPH free radical-scavenging activity assay indicated that the constituents at 17–19 and 22 min on the MS chromatogram showed scavenging activity. Peaks appearing during these times were expected to be active compounds.

### 2.2. Anti-Inflammatory and Antioxidant Activities of Target Components

**Figure 2:**
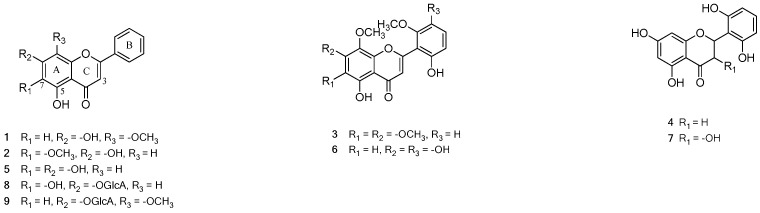
Structure of isolated compounds (**1**–**9**) from *S. baicalensis*.

For confirmation of the LC-MS coupled with a bioassay method, the target compounds were isolated from the MeOH extract. The crude MeOH extract was partitioned into *n*-hexane, EtOAc, *n*-BuOH, and H_2_O layers by liquid–liquid separation. The EtOAc layer was further subjected to column chromatography over silica gel and C_18_-reversed phase silica gel to obtain eight compounds (**1**–**8**). Similarly, the *n*-BuOH layer was successively separated by multicolumn chromatography to yield one compound (**9**). The structures of compounds **1**–**9** were elucidated by ^1^H-NMR, ^13^C-NMR, and MS spectral data, and compared with those reported in the literature. Specifically, they were wogonin (**1**) [18], oroxylin A (**2**) [19], skullcapflavone II (**3**) [20], 2′,5,6′,7-tetrahydroxyflavanone (**4**) [21], baicalein (**5**) [22], visidulin III (**6**) [23], 2′,3,5,6′,7-pentahydroxyflavanone (**7**) [24], baicalin (**8**), and wogonoside (**9**) [25] (Figure 2, Figure S2).

The nine isolated compounds **1**–**9** were evaluated for their NO production–inhibition activity in lipopolysaccharide (LPS)-induced BV2 cells at concentrations of 0.1, 1, and 5 μM (Table 2). NO production inhibitory activity was assessed by the relative NO inhibition rate. At a high concentration of 5 μM, compounds **1**, **8,** and **9** showed strong inhibitory activity with inhibition of 91.4%, 82.2%, and 83.2%, respectively. Compounds **1**, **8**, and **9** featured peaks **m**, **d**, and **j**, respectively. Peaks **m**, **d**, and **j** were consistent with NO inhibitory activity, as seen in Figure 1C.

The nine isolated compounds **1**–**9** were also assessed using DPPH and ABTS free radical-scavenging activity assays (Table 3 and Figure S3 and S4). Among these compounds, **5**, **6**, and **8** demonstrated the highest activity in both the DPPH and ABTS free radical-scavenging activity assays, with peaks **l**, **i**, and **d**. These peaks were in line with the antioxidant activity observed in Figure 1D,E. In particular, baicalin (**8**), which exhibited activity with both bioactivity-screening methods, is well-known for its antioxidant and anti-inflammatory activities, as seen in previous reports [26]. From these results, it was confirmed that our LC-MS coupled with a bioassay method worked well. Also, this new method quickly identified the active compounds from natural products without isolation.

### 2.3. Dereplication through Molecular Networking

**Figure 3:**
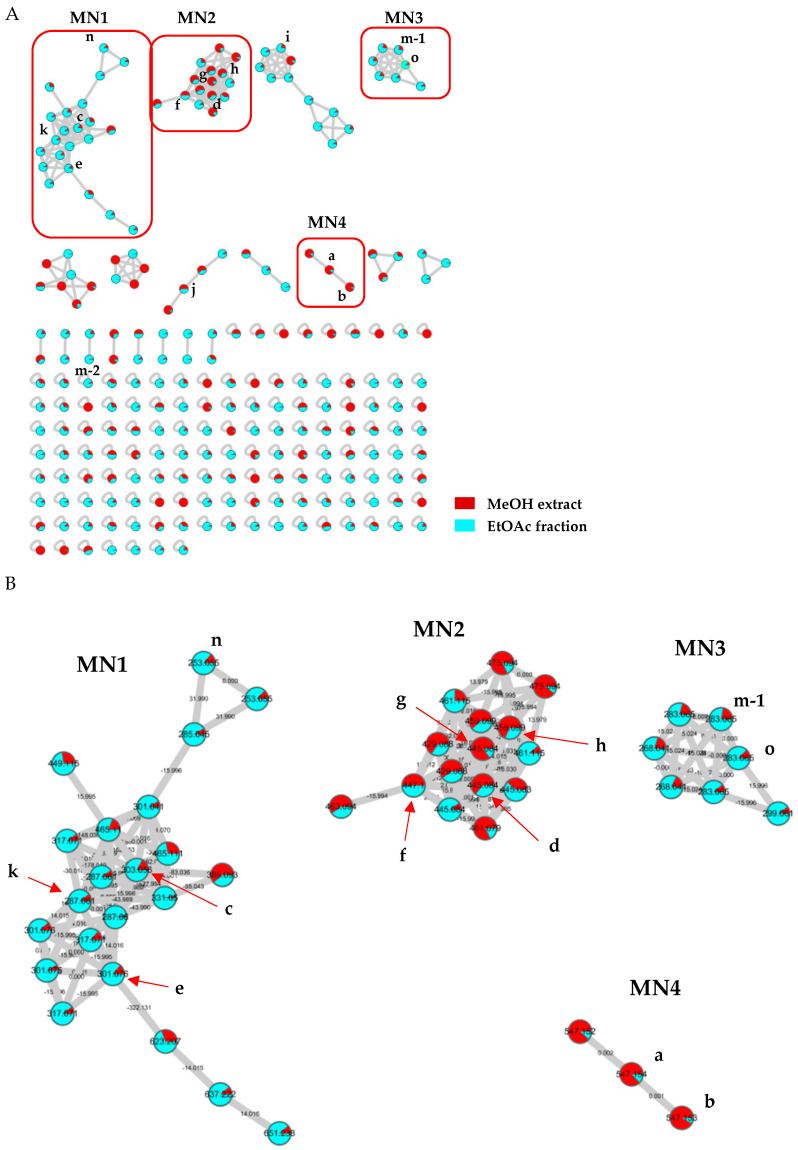
Dereplication using molecular networking. (**A**) Whole molecular network of *S. baicalensis* extract and EtOAc fraction; (**B**) identified peaks and isolated compounds (**a**–**h**, **k** and **m**–**o**) and their clustering.

For dereplication, molecular networking was performed on the MeOH extract and EtOAc fraction of *S. baicalensis,* which contained most of the isolated compounds (Figure 3A). Through clusters containing the identified peaks in the chemical profile, structural similarity was confirmed (Figure 3B). Among many other clusters, molecular network (MN)1 was a cluster in which the overall nodes were included in the EtOAc fraction rather than the MeOH extract. The peaks **c**, **k,** and **n** contained in this MN1 were based on a structure with hydroxyl groups at C-5 and C-7, and peaks **c** and **k** were compounds **7** and **4** (Figure 2), respectively. These structures are flavanones/flavanonols without double bonding in the C ring and are almost similar except for the difference of the presence or absence of a hydroxyl group on C-3. Peaks **d** and **f**–**h**, which occupy the MN2 cluster, all exhibited a value of C_6_H_8_O_6_ missing from the molecular weight in the MS/MS fragments, which was the expressed glucuronide group. Therefore, most of the peaks exhibited a flavone structure. Among the nodes of MN3, peaks **m-1** and **o** were compounds with the same molecular formula but different positions of a methoxy group. Peaks **m-1** and **o** contained a value of CH_3_ missing from the molecular weight in the MS/MS fragments, confirming that the methoxy group was present and its position was different. Peaks **a** and **b** were very similar in structure to sugars linked to chrysin, which is of the flavonoid family, and were confirmed to be in the MN4 cluster. Through these results, it was confirmed that the molecular network was grouped according to structural similarity.

When these molecular networks and LC-MS data coupled with a bioassay method of Figure 1 were shown together, a correlation was observed between the antioxidant and anti-inflammatory components. As seen in Figure 1C–E, 17–20 min showed broad NO production–inhibition and ABTS and DPPH radical inhibitory activities, and the peaks **d**–**j** were distributed in the electrospray ionization (ESI)-MS chromatogram (Figure 1A). The peaks **d** and **f**–**h** of MN2, whose structural similarity was verified through molecular networking, were confirmed. Specifically, peak **i** was in another cluster, but this was a computational result for MS/MS fragments of molecular networking, thereby generating these results. Therefore, it was predicted that the nodes of this MN2 cluster were components with antioxidant and anti-inflammatory activities. Similarly, 24–26 min showed NO production–inhibition activity, expressing a peak m–o in the MS chromatogram. The peaks m and o were distributed in the MN3 cluster, as before. Since the structural similarity of MSMS was confirmed, anti-inflammatory activity was expected from the node of the minor component present in the MN3 cluster.

## 3. Materials and Methods

### 3.1. General Experimental Procedures

Dimethyl sulfoxide (DMSO) and lipopolysaccharide (LPS), dexamethasone, formic acid, 1,1-diphenyl-2-picrylhydrazyl (DPPH), and 2,2′-Azino-bis(3-ethylbenzothiazoline-6-sulfonic acid) (ABTS) were purchased from Sigma-Aldrich. Dulbecco’s modified Eagle’s medium (DMEM) was purchased from Hyclone Laboratories Inc. Fetal bovine serum (FBS), trypsin-ethylenediaminetetraacetic acid (EDTA), and penicillin–streptomycin were purchased from Gibco Industries Inc. High performance liquid chromatography (HPLC)-grade water and acetonitrile were obtained from Fisher Scientific Korea Ltd. DMSO-*d_6_* and methanol-*d_4_* were purchased from Cambridge Isotope Laboratories Inc. The Griess reagent for the measurement of NO production was generated from 1% sulfanilamide (Sigma-Aldrich), 0.1% naphthylethylenediamine dihydrochloride (Sigma-Aldrich), and 2% phosphoric acid (Wako Pure Chemical Industries). The water soluble tetrazolium (WST) kit (EZ-cytox) for the cell viability assay was purchased from Daeil Lab Service Co. NMR spectra were recorded on a Bruker Avance DPX 300 and Bruker Avance III 600. The ESI-MS spectra were measured with an Agilent 6530 Q-TOF mass spectrometer (Agilent, Santa Clara, USA).

### 3.2. Plant Material

The root of *S. baicalensis* was purchased from Dongwoodang Pharmaceutical Corporation (Youngcheon, Republic of Korea) and identified by Dr. Ki Yong Lee, a professor at the College of Pharmacy, Korea University. A voucher specimen (KUP-HD006) was deposited at the Laboratory of Pharmacognosy, College of Pharmacy, Korea University.

### 3.3. Extraction and Isolation

Dried root of *S. baicalensis* (1.5 kg) was extracted with 80% MeOH three times under ultrasonication for 60 min. The 80% MeOH solution was filtered and concentrated in vacuum at 40 °C. The obtained residue (170.1 g) was suspended in H_2_O (800 mL) and partitioned with *n*-hexane (800 mL × 3, 1.5 g), EtOAc (800 mL × 3, 15.7 g), and *n*-BuOH (800 mL × 3, 17.8 g). The EtOAc fraction was then subjected to silica gel (0.040–0.063 mm, 230–400 mesh) column chromatography (200 g, 6 × 50 cm) with a gradient of *n*-hexane-EtOAc (100:0 to 0:100; 1 L of each solvent) to yield nine fractions (E1–E9). Fraction E2 (730 mg) was recrystallized to yield compound **1** (428 mg). E3 (1.5 g) was further chromatographed by medium-pressure liquid chromatography (MPLC) on a silica gel column (Biotage SNAP ultra 100 g, 1.5 × 16 cm, 55-μm particle size) with dichloromethane (DCM)-MeOH (100:0 to 90:10, 45 mL/min) to yield nine subfractions (E3.1–3.9). Compounds **2** (6 mg), **3** (201 mg), and **4** (190 mg) were purified by recrystallization from E3.1 (53 mg), 3.4 (320 mg), and 3.5 (350 mg), respectively. Compound **5** (1115 mg) was purified E4 and 6 (2.3 g) by recrystallization. E5 (1.2 g) was rechromatographed with medium performance liquid chromatography (MPLC) (Biotage SNAP ultra 100 g, 1.5 × 16 cm, 55-μm particle size) with DCM-MeOH (100:0 to 90:10, 40 mL/min) to afford nine subfractions (E5.1–5.9). Compound **6** (56 mg) was obtained from E5.4 (153 mg). E5.6 (195 mg) was recrystallized to yield compound **7** (23 mg). The *n*-BuOH fraction was subjected to Diaion HP-20 (250–850 μm, 6 × 80 cm) column chromatography with a gradient of H_2_O-MeOH (100:0 to 0:100; 1 L of each solvent) to yield six fractions (B1–B6). B4 (3.2 g) was subjected to silica gel column chromatography (80 g, 3.5 × 25 cm) with a gradient of CHCl_3_-MeOH-H_2_O (25:4:1 to 6:5:1; 600 mL of each solvent) to yield 23 fractions (B4.1–4.23). Compound **8** (320 mg) was purified by recrystallization from B4.21 (480 mg). B4.19 (840 mg) was subjected to reversed-phase C_18_ column chromatography (200 g, 3 × 70 cm, 30–50 μm) with a gradient of MeOH-H_2_O (1:2 to 1:1; 500 mL of each solvent) to yield 10 fractions (B4.19.1–4.19.10). Compound **9** (5 mg) was purified from B4.19.5 (50 mg) by recrystallization.

### 3.4. LC-QTOF MS/MS Coupled with Bioassay

The LC-QTOF MS/MS coupled with a bioassay method was composed of two phases. The first received the chemical profile of the sample by analysis with LC-QTOF MS/MS. The second phase was based on collecting the eluent through the column for 30 s per well with a 96-well plate. HPLC analysis was carried out a on Shiseido CapCell PAK C18 column particle size 5 μm (150 × 4.6 mm) using Agilent 126 series system. The mobile phase consisted of water with 0.1% formic acid (solvent A) and acetonitrile with 0.1% formic acid (solvent B), which were applied with the following gradient elution: 5% B (0–5 min) and 5–95% B (5–30 min). The first injection was 5 µL of *S. baicalensis* MeOH extract injected at 1 mg/mL and the second injection was 20 µL of the extract injected at 25 mg/mL. The flow rate was 0.6 mL/min. A UV chromatogram was recorded at 254 nm. In addition, an Agilent 6530 Q-TOF mass spectrometer (Agilent, Santa Clara, USA) was connected to an HPLC system via an electrospray ionization (ESI) interface and ionized in negative mode. The MS scan range was 50–1700 *m*/*z*. The MS/MS fragmentation was set at collision energies of 10, 20, and 30 eV. Data acquisition and processing were carried out by Mass Hunter Workstation software LC/MS Data Acquisition for 6530 series QTOF (version B.05.00). The collected sample in the plate was dried by a vacuum oven (JEIO Tech, OV-12) at 40 °C for 12 h to completely remove the mobile phase before assaying.

#### 3.4.1. NO Production Inhibitory Assay and Cell Viability

BV2 mouse microglia cells were obtained from the College of Pharmacy and Research Institute of Pharmaceutical Science, Seoul National University, Seoul, Korea. Cells were maintained in DMEM supplemented with 10% FBS and 1% penicillin–streptomycin at 37 °C in a humidified incubator under 5% CO_2_ and 95% air. BV2 cells (2 × 10^5^ cells/mL) were seeded in 96-well plates and cultured for 23 h. The dried samples collected through LC/MS were dissolved in 35 μL of DMEM without FBS. After the medium was replaced with serum-free DMEM, the cells were pretreated with samples for 1 h. Next, LPS (100 ng/mL) was added and the cells were incubated for 24 h. A total of 100 μL of supernatant in each well was mixed with 100 μL of Griess reagent (1% sulfanilamide, 0.1% naphthylethylenediamine dihydrochloride, 2% phosphoric acid). After incubation for 10 min at room temperature, the absorbance at 550 nm was measured using a spectrophotometer. NO production inhibitory activity for each well was calculated as follows: Inhibition activity (%) = 100 × (OD of LPS treated cultures − OD of LPS sample-treated cultures)/(OD of LPS-treated cultures − OD of control cultures). For cell viability, 10 μL of WST solution was added to 100 μL of the remaining supernatant. The plate was incubated for 2 h and the absorbance at 450 nm was measured. Cell viability for each well was calculated as follows: 100 × (OD of LPS sample-treated cultures)/(OD of control cultures).

#### 3.4.2. DPPH and ABTS Free Radical-Scavenging Assay

Antioxidant activity assay was carried out using a DPPH and ABTS assay. First, the DPPH assay was employed to measure the DPPH free radical-scavenging ability. Solutions of the collected sample through LC/MS were prepared in ethanol at a concentration of 300 µL per well. A total of 10 µL of dissolved sample was added to 190 µL of 15 µM DPPH solution in ethanol. Thereafter, the mixture was incubated in the dark at room temperature. After incubation for 30 min, absorbance at 517 nm was measured using a spectrophotometer. The inhibition of the radical-scavenging activity for each sample was calculated as follows: Inhibition activity (%) = 100 − [(S − S_0_)/(C − C_0_)] × 100, where C and S were the absorbance of the control and inhibitor after 30 min and S_0_ and C_0_ were the absorbance of the control and inhibitor in ethanol without DPPH solution.

The ABTS free radical was produced by a chemical oxidation reaction with potassium persulfate. A measured amount of 2.5 mM of ABTS was mixed with an equal volume of 2.45 mM potassium persulfate and vortexed for 30 s. Then, 100 µL of the dissolved sample was added to 200 µL of ABTS solution. The mixture was then incubated in the dark at room temperature. After incubation for 10 min, the absorbance at 718 nm was measured with a spectrophotometer. The inhibition of the radical-scavenging activity for each sample was calculated as follows: Inhibition activity (%) = 100 − [(S − S_0_)/(C − C_0_)] × 100, where C and S were the absorbance of the control and inhibitor, respectively, after 30 min, while S_0_ and C_0_ were the absorbance of the control and inhibitor, respectively, in distilled water without ABTS solution.

### 3.5. Molecular Networking

The total extract and EtOAc fraction were analyzed using molecular networking, a dereplication technique utilizing tandem MS data. Molecular networking was conducted on the Global Natural Products Social Molecular Networking (GNPS) web platform (https://gnps.ucsd.edu). The MS/MS data were converted to mzXML format using MS Convert software (Microsoft, Redmond, USA) and uploaded to GNPS using filezila software. The molecular network was created by uploading the MS/MS fragmentation similarity calculation under the following parameters: The precursor ion mass tolerance was *m*/*z* 0.02 Da, the fragment ion mass tolerance was *m*/*z* 0.02 Da, the minimum cosine score was 0.7, the minimum matched fragment ion was 4, the minimum cluster size was 2, the generated molecular network was schematically illustrated using Cytoscape 3.6.0 software, and dereplication was performed using MS/MS patterns of each cluster. The relevant molecular networking results can be accessed via the following link: https://gnps.ucsd.edu/ProteoSAFe/status.jsp?task=521d25bff0bb481e94326c5225c91b5f

## 4. Conclusions

In conclusion, this study applied an LC-MS coupled with a bioassay method to target anti-inflammatory and antioxidant components from *S. baicalensis* more easily than previous bioassay-guided purification methods. In addition, structural similarity was confirmed with molecular networking as the dereplication identifier for the main constituents of *S. baicalensis*. Thus, the targeted components exhibited strong activity compared to other compounds. A correlation was shown between the components showing antioxidant and anti-inflammatory activity through LC-MS coupled with a bioassay method and molecular networking. Other nodes around the peak with demonstrated activity according to structural similarity were confirmed through molecular networking, showing the possibility of applying to other natural products. Therefore, these findings demonstrate that there is a foundation of future investigations in targeting and identifying bioactive components more easily and quickly using LC-MS coupled with a bioassay and molecular networking as part of dereplication.

## Figures and Tables

**Table 1:** LC-MS analysis of constituents in the MeOH extract of *S. baicalensis* in Figure 1.

Peak No.	Expected Compounds	t_R_ (min)	Observed *m*/*z*	Calculated *m*/*z*	Molecular Formula [M − H]^−^	MS/MS Fragments (*m*/*z*)	UV (λ max, nm)	Isolated Compounds
**a**	Chrysin-6-C-ara-8-C-glu	15.096	547.1565	547.1457	C_26_H_27_O_13_	337 [M-C_11_H_14_O_4_-H]^−^	273, 314	
**b**	Chrysin-6-C-glu-8-C-ara	15.471	547.1559	547.1457	C_26_H_27_O_13_	337 [M-C_11_H_14_O_4_-H]^−^	273, 314	
**c**	2′,3,5,6′,7-Pentahydroxyflavanone	16.595	303.0572	303.0510	C_15_H_11_O_7_	125 [M-C_9_H_6_O_4_-H]^−^	285	**7**
**d**	Baicalin	17.657	445.0859	445.0776	C_21_H_17_O_11_	269 [M-C_6_H_8_O_6_-H]^−^	278, 315	**8**
**e**	Unidentified flavonoid	18.094	301.0774	301.0718	C_16_H_13_O_6_	139 [M-C_9_H_6_O_3_-H]C^−^	-	
**f**	Dihydrobaicalin	18.281	447.1014	447.0933	C_21_H_19_O_11_	271 [M-C_6_H_8_O_6_-H]^−^	285	
**g**	Baicalein-6-glucuronide	18.469	445.0851	445.0776	C_21_H_17_O_11_	269 [M-C_6_H_8_O_6_-H]^−^	280	
**h**	Oroxylin A-7-glucuronide	18.593	459.1016	459.0933	C_22_H_19_O_11_	283 [M-C_6_H_8_O_6_-H]^−^	273, 314	
**i**	Viscidulin Ⅲ	18.843	345.0680	345.0616	C_17_H_13_O_8_	315 [M-CH_2_O-H]^−^	265	**6**
**j**	Wogonoside	19.093	459.1013	459.0933	C_22_H_19_O_11_	283 [M-C_6_H_8_O_6_-H]^−^	274	**9**
**k**	2′,5,6′,7-Tetrahydroflavanone	19.718	287.0617	287.0561	C_15_H_11_O_6_	125 [M-C_9_H_6_O_3_-H]^−^	-	**4**
**l**	Baicalein	21.779	269.0511	269.0455	C_15_H_9_O_5_	195 [M-C_6_H_2_-H]^−^	275, 323	**5**
**m**	Wogonin/Skullcapflavone Ⅱ	24.402	283.0670/373.1001	283.0612/373.0929	C_16_H_11_O_5_/C_19_H_17_O_8_	268 [M-CH_3_-H]^−^ /343 [M-CH_2_O-H]^−^	275	**1/3**
**n**	Chrysin	24.590	253.0561	253.0506	C_15_H_9_O_4_	143 [M-C_6_H_6_O_2_-H]^−^	-	
**o**	Oroxylin A	25.027	283.0673	283.0612	C_16_H_11_O_5_	268 [M-CH_3_-H]^−^	-	**2**

**Table 2:** Effect of isolated compounds on NO production in BV2 cells.

Compounds	Concentration (mM)	Relative NO Inhibition (%) ^1^	Viability (%)
Control		100.0 ± 0.0 **	100.0 ± 1.6
LPS	100 ng/mL	0.0 ± 1.4	102.4 ± 1.9
Dexamethasone ^2^	10	57.4 ± 1.7 ***	98.4 ± 0.5
1	0.1	67.5 ± 0.4 **	106.4 ± 0.4
	1	72.5 ± 1.8 *	111.7 ± 0.1
	5	91.4 ± 0.1 **	110.6 ± 2.3
2	0.1	60.5 ± 4.2 *	112.2 ± 3.3
	1	58.7 ± 3.6 **	111.0 ± 3.2
	5	69.7 ± 1.0 **	101.1 ± 3.2
3	0.1	57.4 ± 1.0 **	115.7 ± 3.1
	1	56.7 ± 0.7 **	108.0 ± 1.3
	5	70.4 ± 0.3 **	116.4 ± 0.2
4	0.1	59.5 ± 0.8 **	105.5 ± 0.8
	1	61.8 ± 1.8 **	103.5 ± 1.1
	5	66.9 ± 0.9 **	104.1 ± 1.8
5	0.1	61.6 ± 0.1 **	99.2 ± 0.4
	1	65.6 ± 2.3 **	97.0 ± 2.3
	5	71.3 ± 0.3 **	100.4 ± 0.2
6	0.1	63.1 ± 0.4 **	102.4 ± 1.6
	1	60.0 ± 1.8 **	102.2 ± 0.1
	5	68.8 ± 0.2 **	101.8 ± 0.3
7	0.1	62.3 ± 3.2 *	113.9 ± 1.3
	1	60.7 ± 1.6 **	112.4 ± 1.9
	5	62.5 ± 1.0 *	111.4 ± 3.6
8	0.1	62.5 ± 0.4 **	114.8 ± 2.0
	1	63.0 ± 0.0 **	116.3 ± 2.2
	5	82.2 ± 1.0 **	109.3 ± 1.6
9	0.1	63.9 ± 2.4 *	117.7 ± 1.6
	1	66.6 ± 1.0 **	117.8 ± 0.4
	5	83.2 ± 0.9 **	108.9 ± 0.7

^1^ Sample-treated differs significantly from LPS-treated, * *p* < 0.05, ** *p* < 0.01, *** *p* < 0.001; ^2^ Dexamethasone was used as a positive control for NO production in BV2 cells.

**Table 3:** Effect of isolated compounds on DPPH and ABTS free radical-scavenging activity.

Compounds	DPPH	ABTS
	IC50 ^1^ (μM)	
**1**	>50	>50
**2**	>50	>50
**3**	>50	>50
**4**	>50	>50
**5**	17.0 ± 1.7	16.5 ± 0.5
**6**	16.4 ± 1.6	15.3 ± 0.5
**7**	>50	>50
**8**	15.1 ± 0.8	10.8 ± 0.8
**9**	>50	>50
Trolox ^2^	38.1 ± 1.0	12.8 ± 0.3

^1^ IC_50_ = The half-maximal inhibitory concentration; ^2^ Trolox was used as a positive control for DPPH and ABTS free radical-scavenging activity.

## Supplementary Materials

The following are available online. Figure S1: MS, MS/MS, UV spectra of each peak in Table 1; Figure S2: ^1^H and ^13^C NMR spectrum of each isolated compounds **1**–**9**; Figure S3: Sigmoidal dose–response curves of compounds on DPPH radical-scavenging activity, Figure S4: Sigmoidal dose-response curves of compounds on ABTS radical-scavenging activity.
