# Supplementary material for: Characterization of Anti-Inflammatory and Antioxidant Constituents from Scutellaria baicalensis Using LC-MS Coupled with a Bioassay Method
Source: Molecules. 2020 Aug 9;25(16):3617. doi: 10.3390/molecules25163617 (PMC7464942; doi:10.3390/molecules25163617)

**Supporting information**

# Characterization of Anti-Inflammatory and Antioxidant Constituents from *Scutellaria baicalensis* using LC-MS Coupled with a Bioassay Method

**Yoo Kyong Han [1], Hyunwoo Kim [2], Hyeji Shin [1], Jiyeon Song [1], Mi Kyeong Lee [3], Byoungduck Park [4,*] and Ki Yong Lee [1,*]**

[1] College of Pharmacy, Korea University, Sejong 30019, Republic of Korea; yookyong05@korea.ac.kr (Y.K.H.); hjshin90@korea.ac.kr (H.S.); sjy777777@naver.com (J.S.)
[2] Center for Marine Biotechnology and Biomedicine, Scripps Institution of Oceanography, La Jolla, CA 92093, United States; hwkim8906@gmail.com (H.K.)
[3] College of Pharmacy, Chungbuk National University, Cheongju 28160, Republic of Korea; mklee@chungbuk.ac.kr (M.K.L)
[4] College of Pharmacy, Keimyung University, Daegu 42601, Republic of Korea
* Correspondence: bdpark@kmu.ac.kr (B.P.); kylee11@korea.ac.kr (K.Y.L.); Tel.: +82-53-580-6653 (B.P.); Tel.: +82-44-860-1623 (K.Y.L)

**Figure 1S** MS, MS/MS, UV spectra of each peak in Table 1

**MS spectrum (*m/z*)** | **UV spectrum** | **MS/MS spectrum (*m/z*)**

Peak **a**

Peak **b**

Peak **c**

Peak **d**

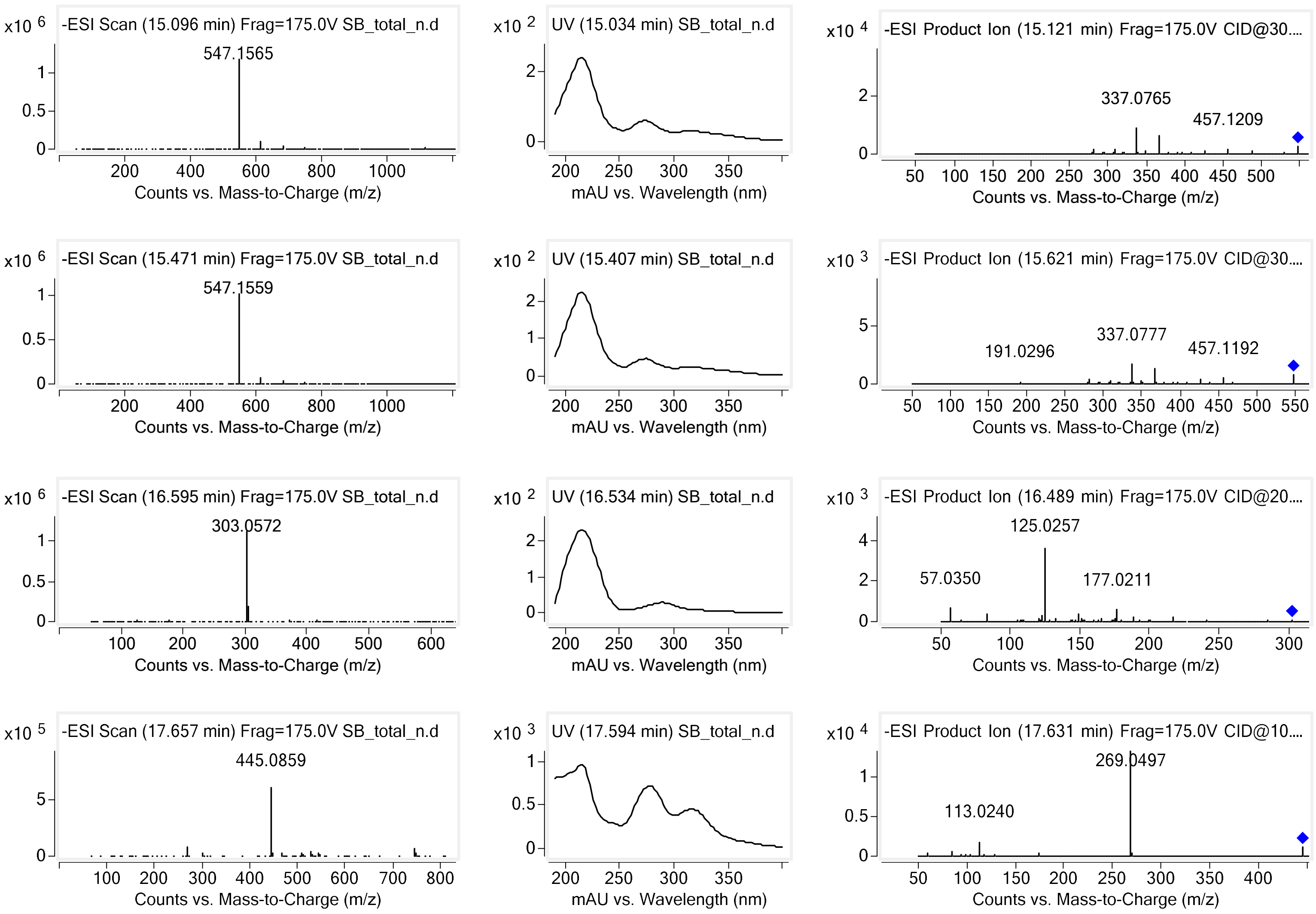

Peak **e**

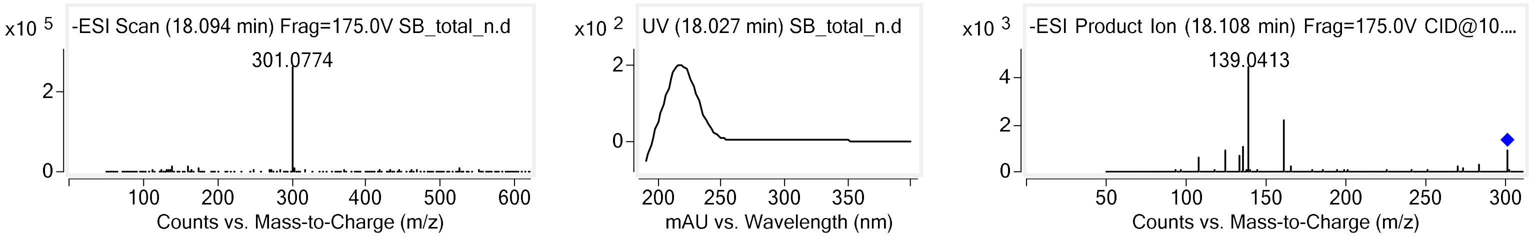


Peak **f**

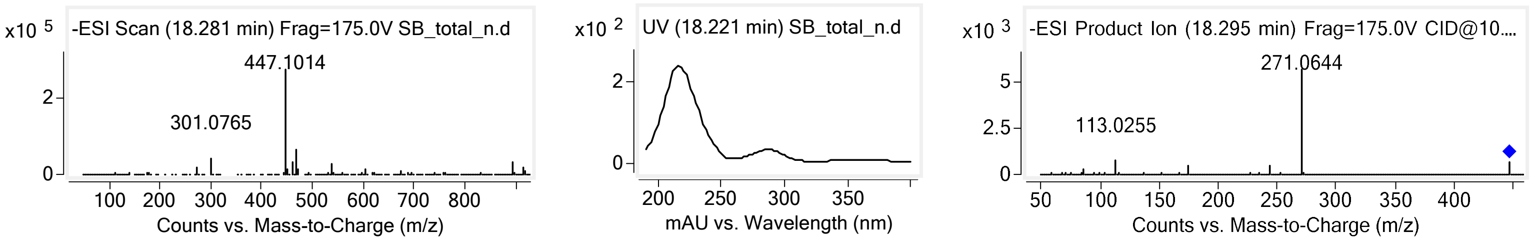


Peak **g**

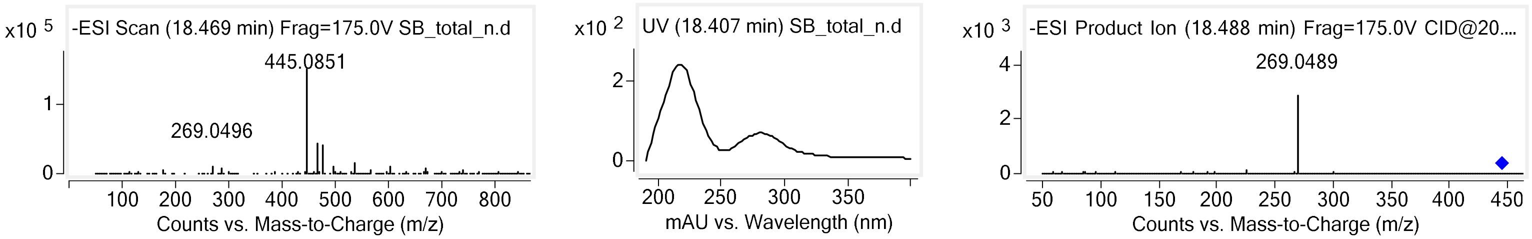


Peak **h**

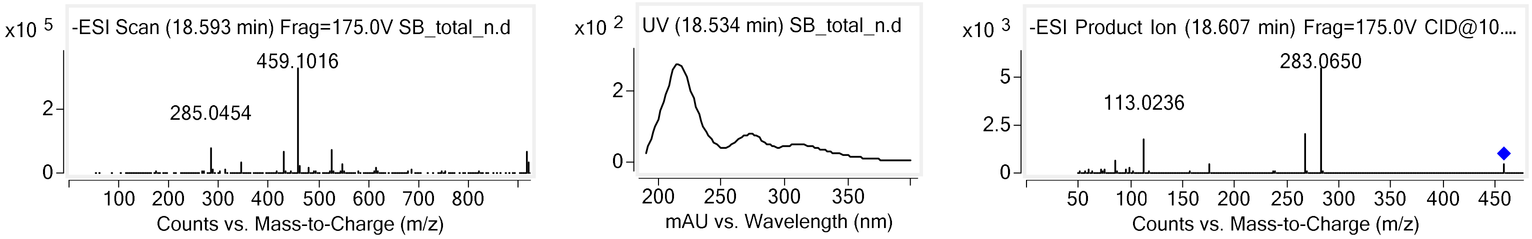

Peak **i**

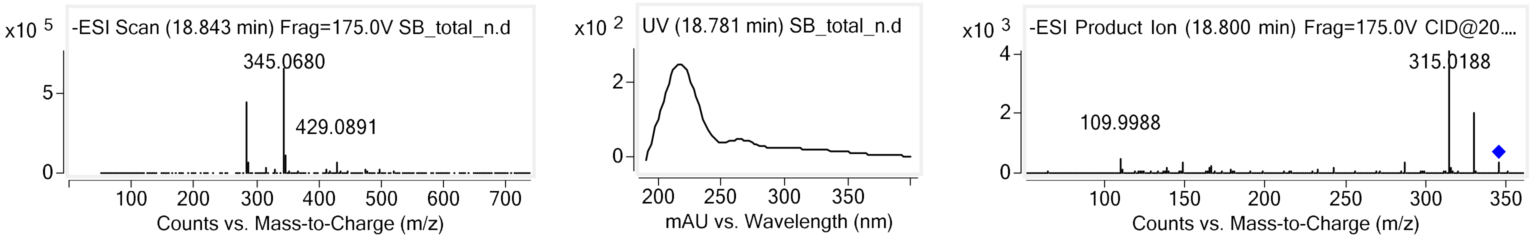


Peak **j**

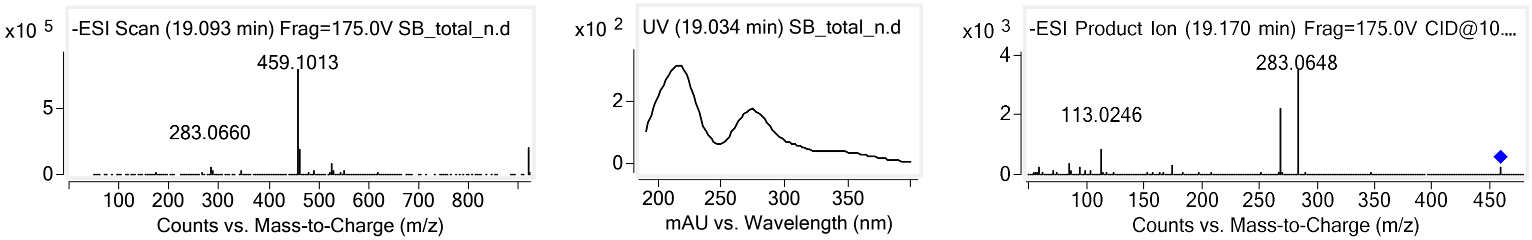


Peak **k**

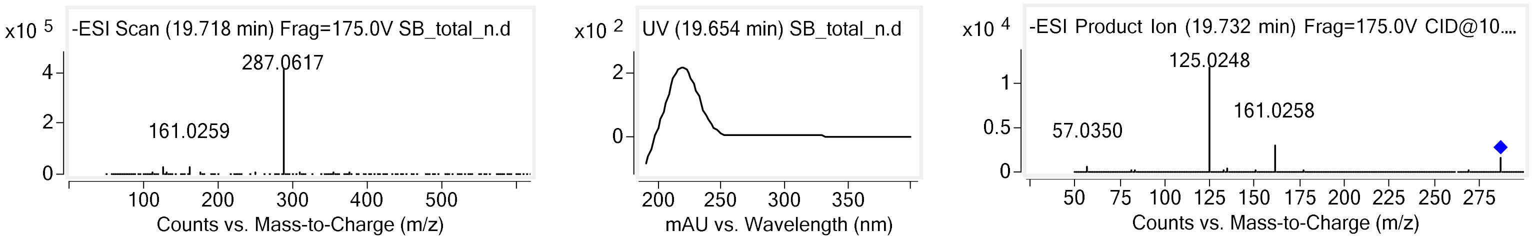


Peak **l**

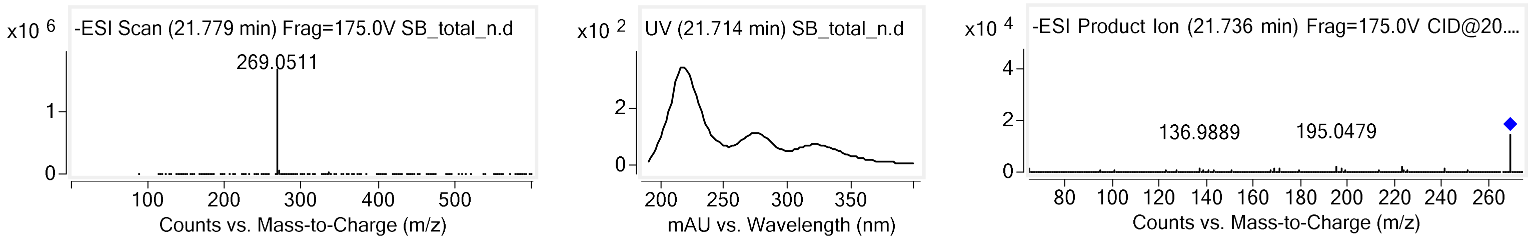

Peak **m**

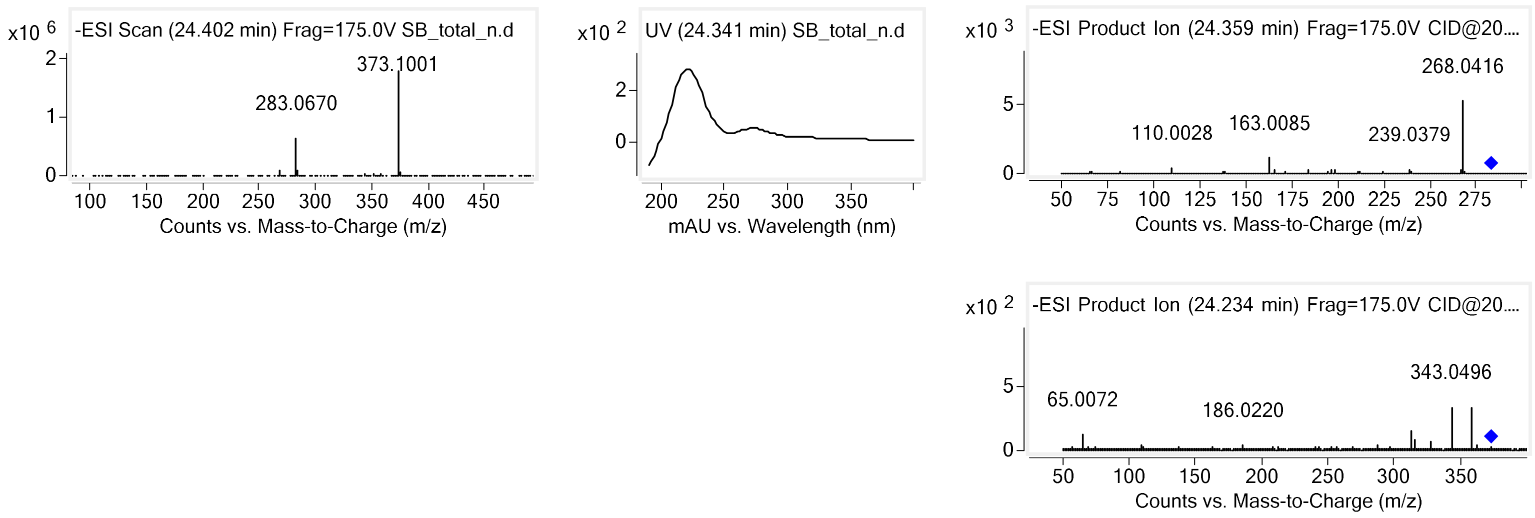


Peak **n**

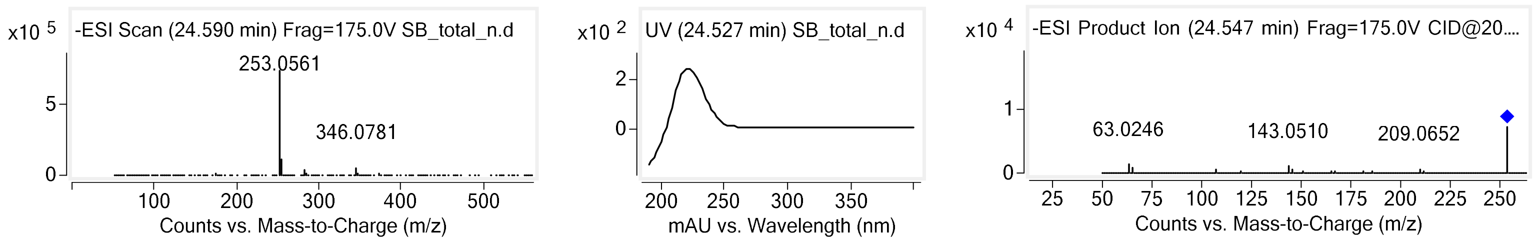


Peak **o**

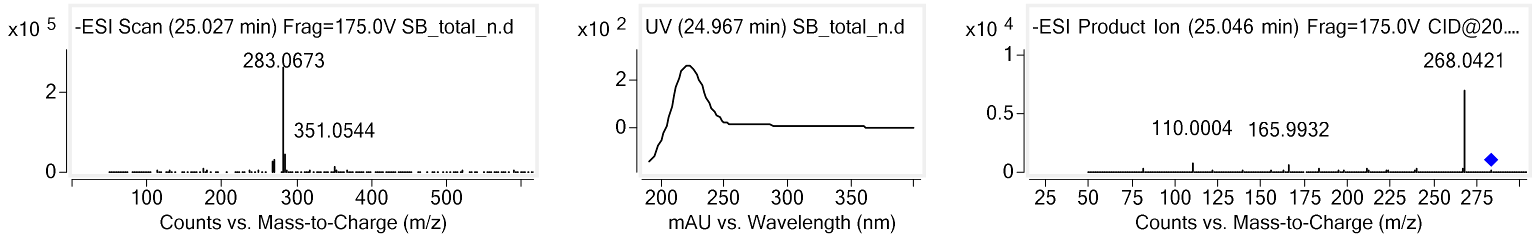

**Figure 2S** $^{1}H$ and $^{13}C$ NMR spectra of each isolated compounds **1-9**

$^{1}H$ NMR spectrum of compound **1** (DMSO-$d_6$, 300 MHz)

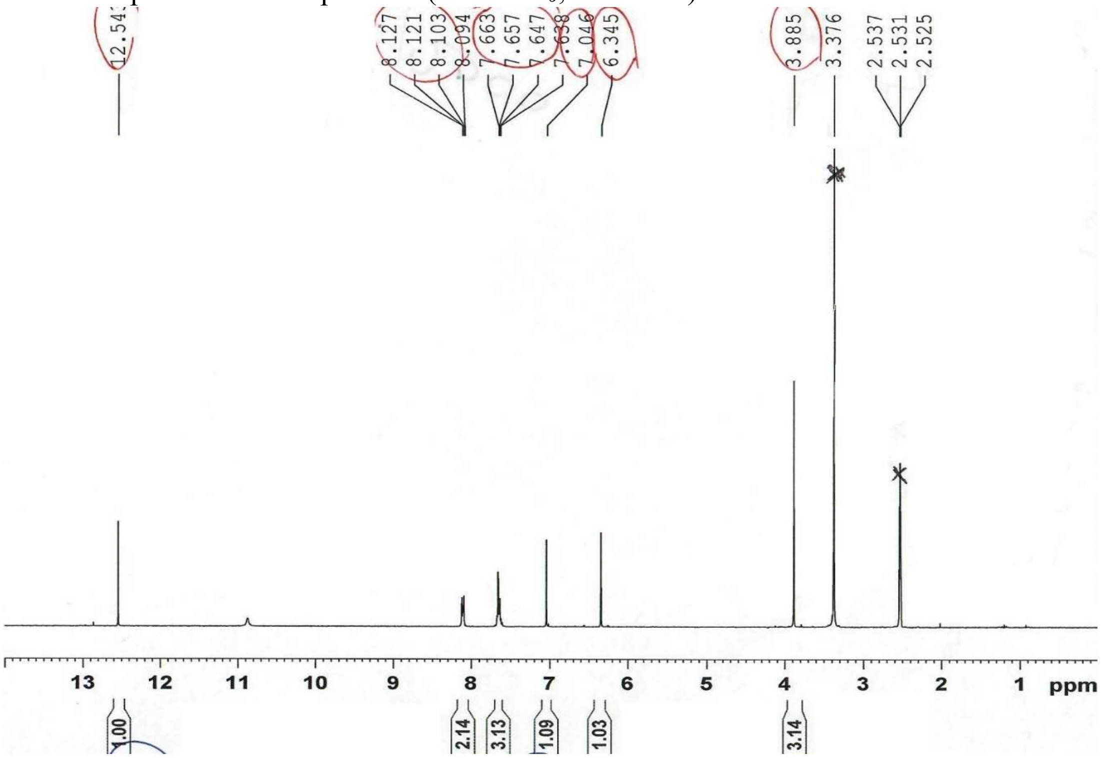

$^{13}C$ NMR spectrum of compound **1** (DMSO-$d_6$, 75 MHz)

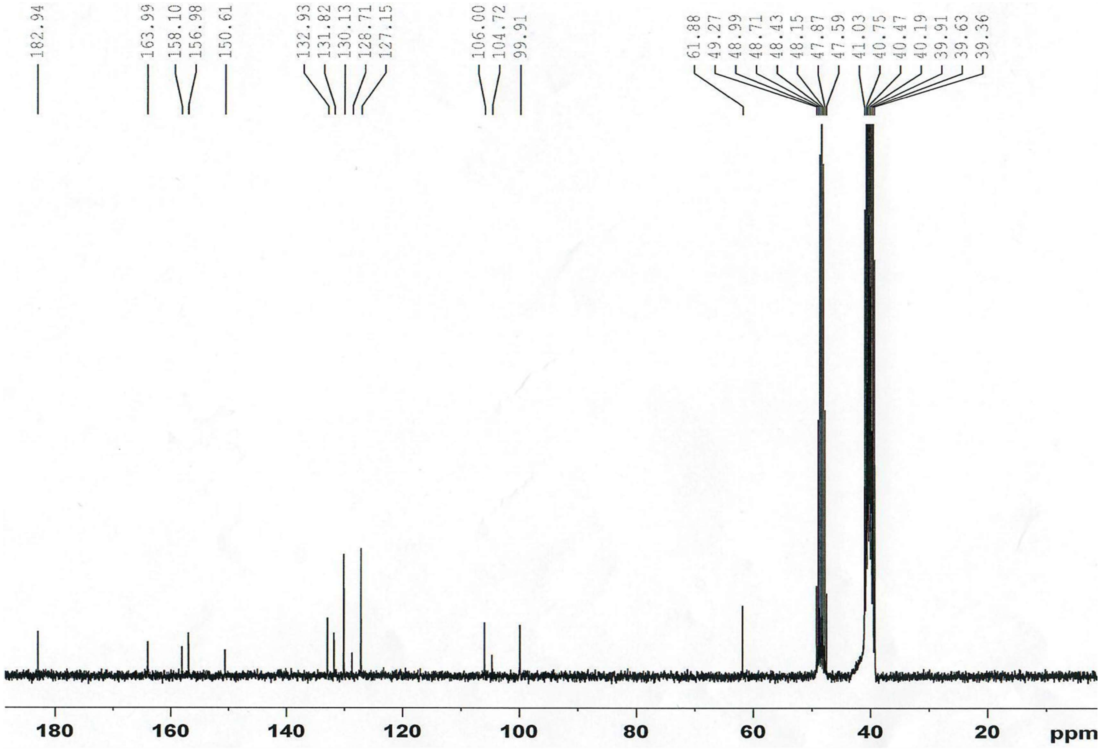

$^{1}$H NMR spectrum of compound **2** (Acetone-$d_6$, 600 MHz)

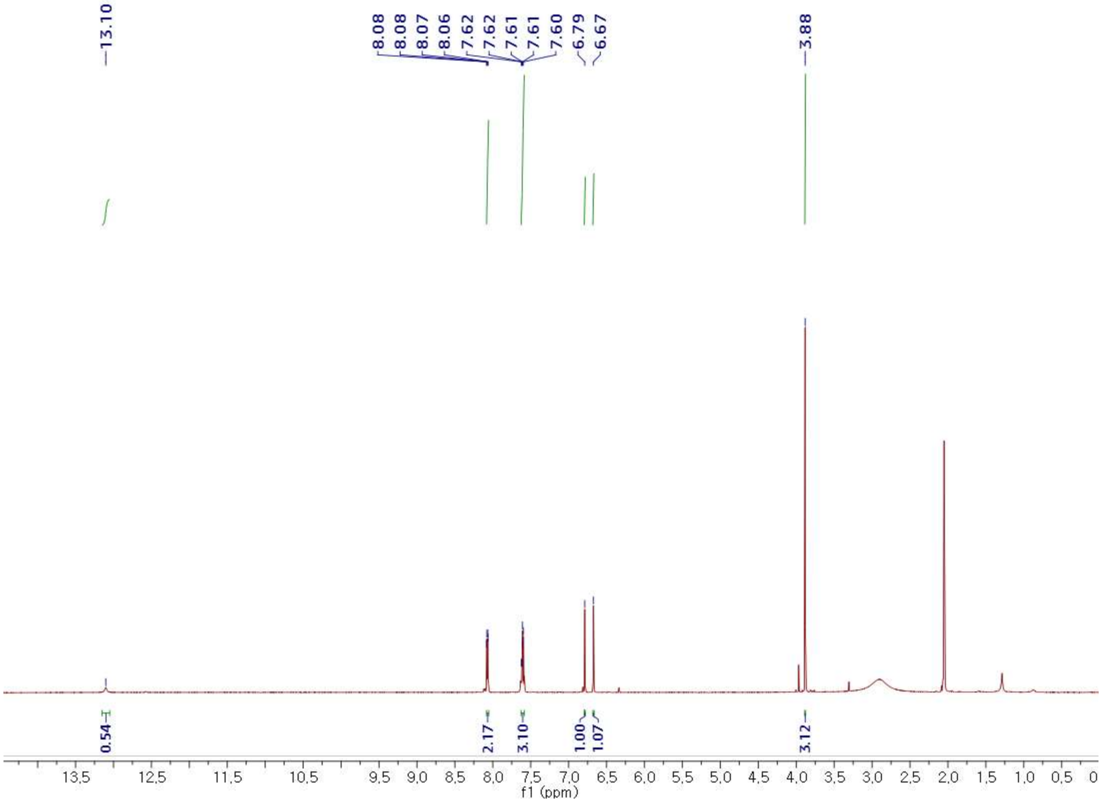


$^{13}$C NMR spectrum of compound **2** (Acetone-$d_6$, 125 MHz)

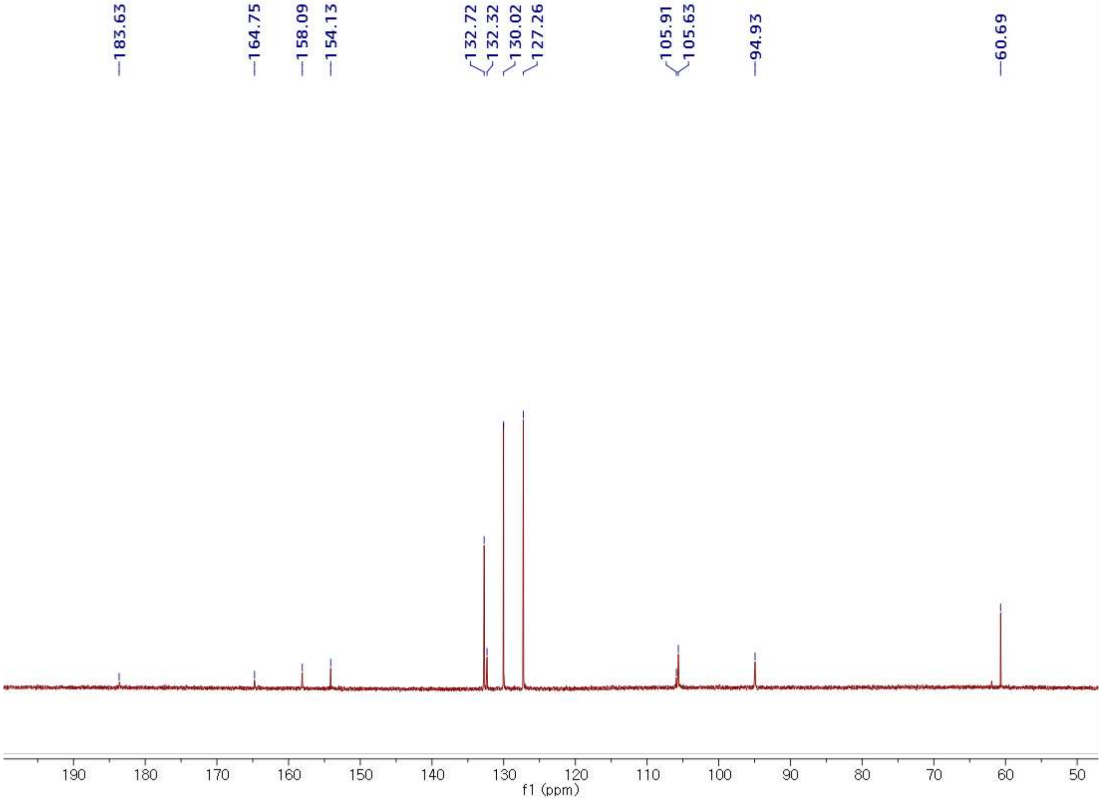

$^{1}$H NMR spectrum of compound **3** (Chloroform-*d*, 600 MHz)

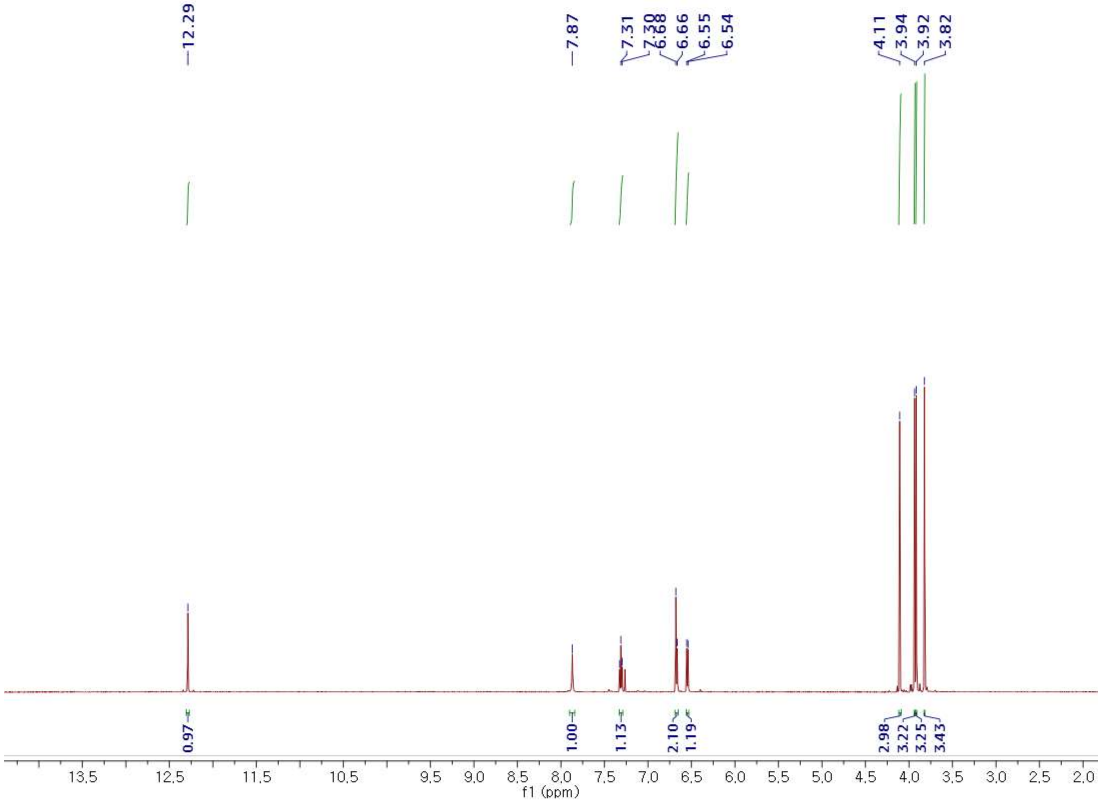


$^{13}$C NMR spectrum of compound **3** (Chloroform-*d*, 125 MHz)

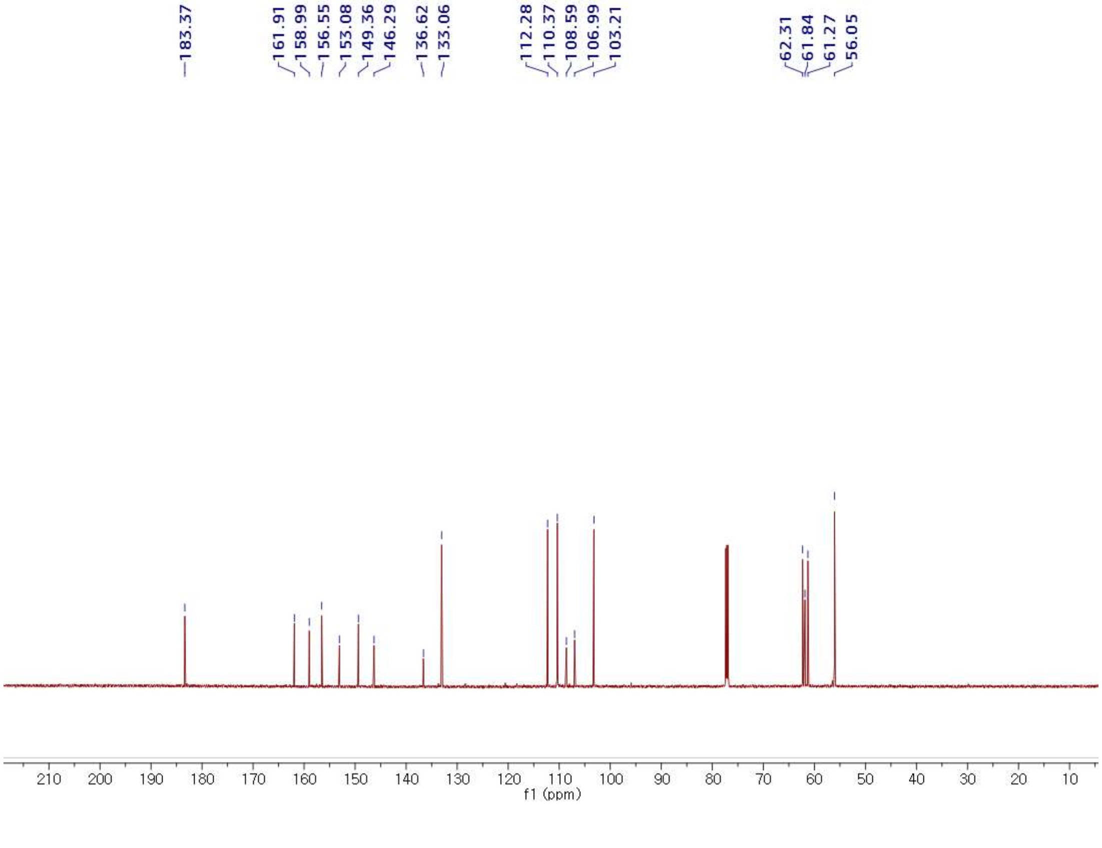

$^{1}$H NMR spectrum of compound **4** (Methanol-$d_4$, 300 MHz)

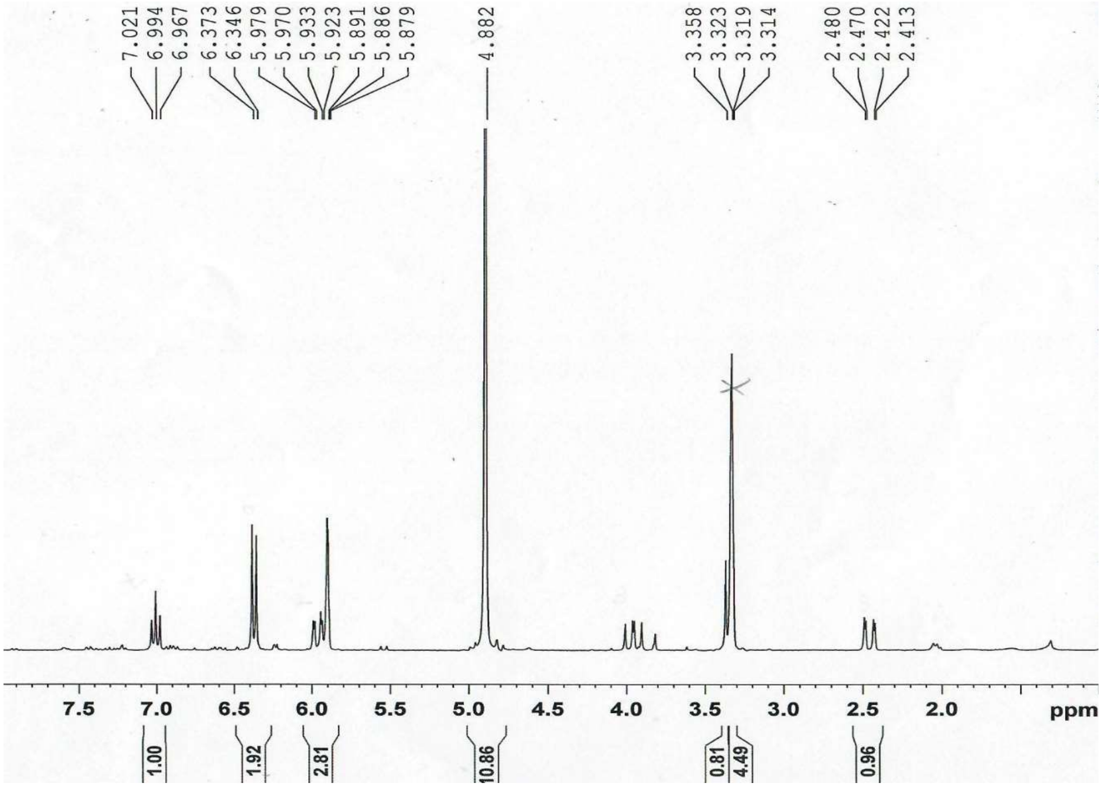


$^{13}$C NMR spectrum of compound **4** (Methanol-$d_4$, 75 MHz)

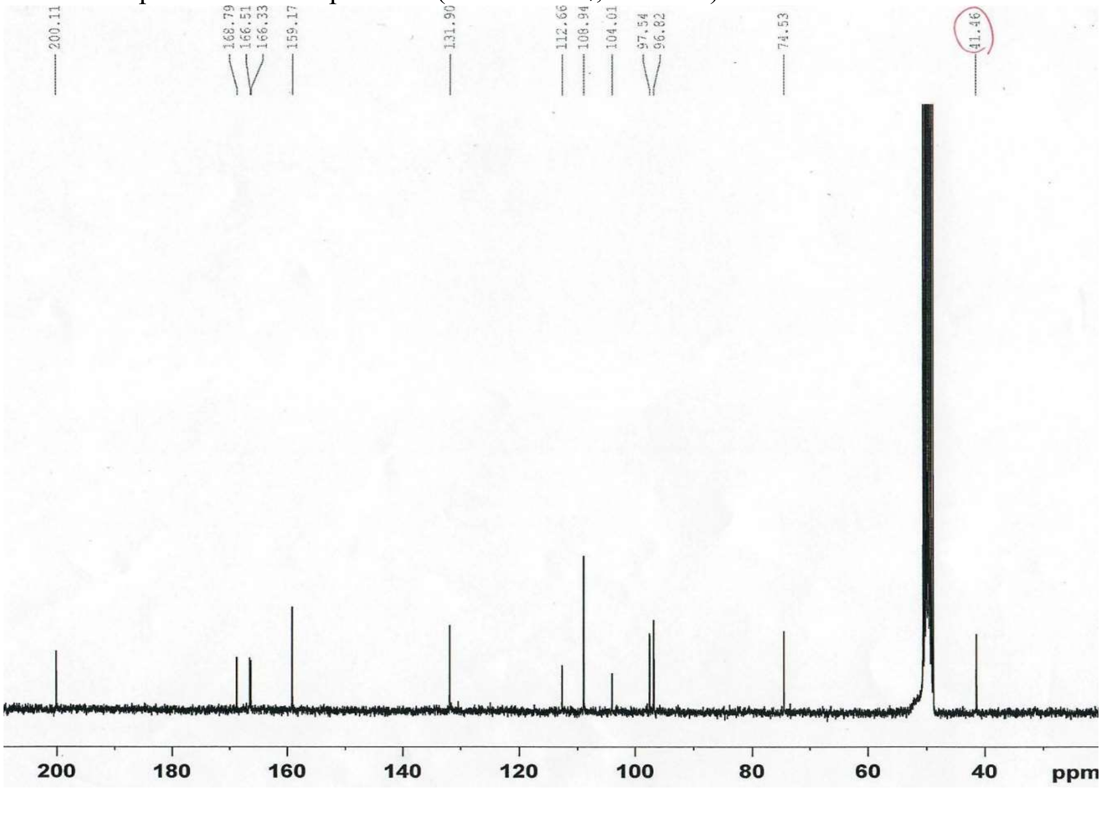

$^1H$ NMR spectrum of compound **5** (DMSO-$d_6$, 600 MHz)

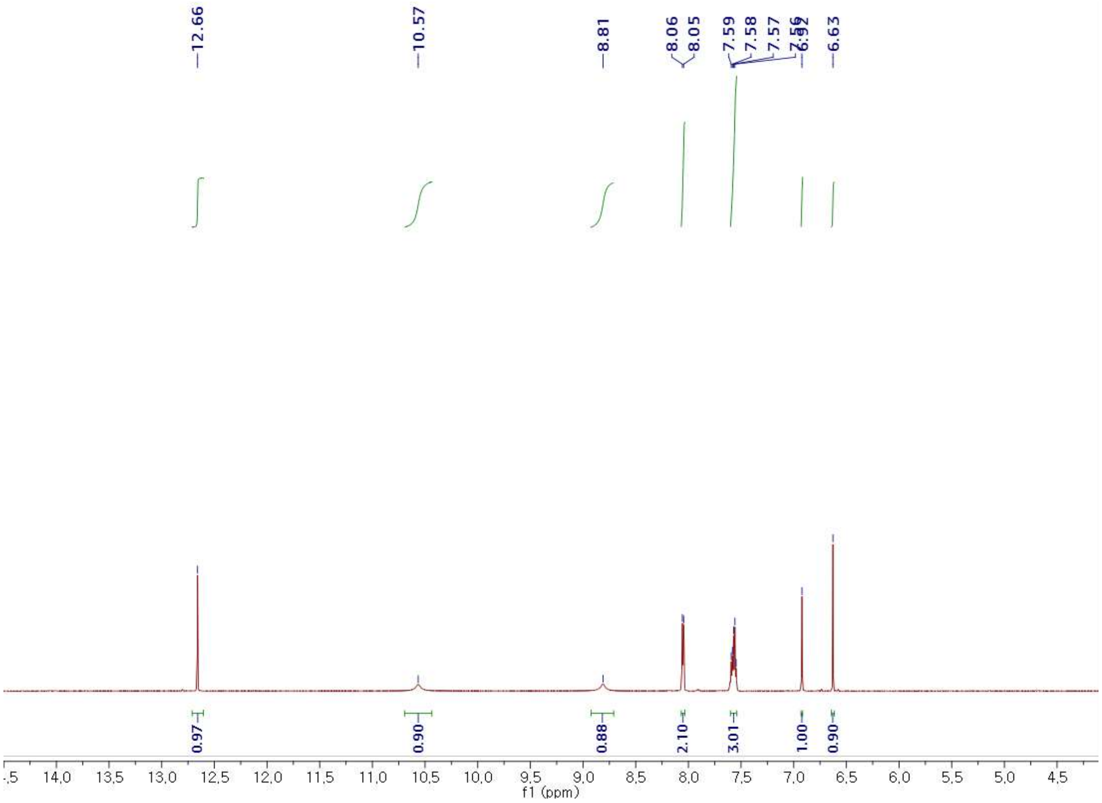


$^{13}C$ NMR spectrum of compound **5** (DMSO-$d_6$, 125 MHz)

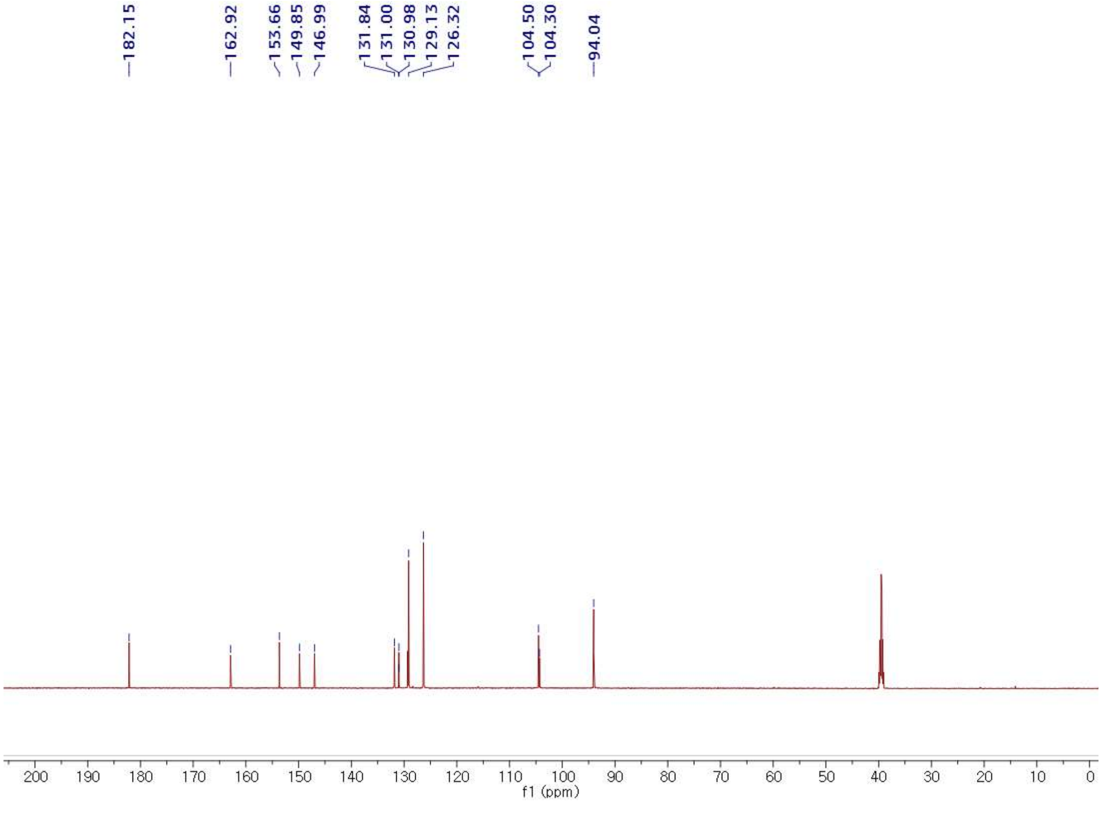

$^{1}$H NMR spectrum of compound **6** (Methanol-$d_4$, 300 MHz)

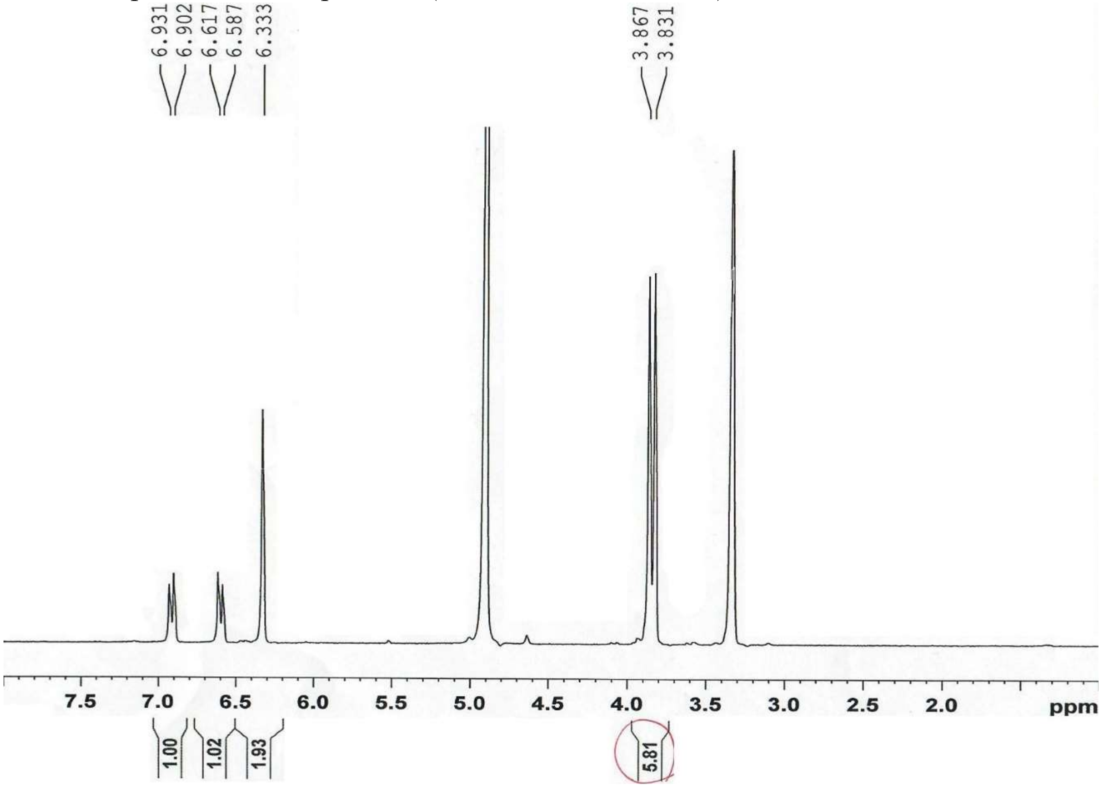


$^{13}$C NMR spectrum of compound **6** (Methanol-$d_4$, 75 MHz)

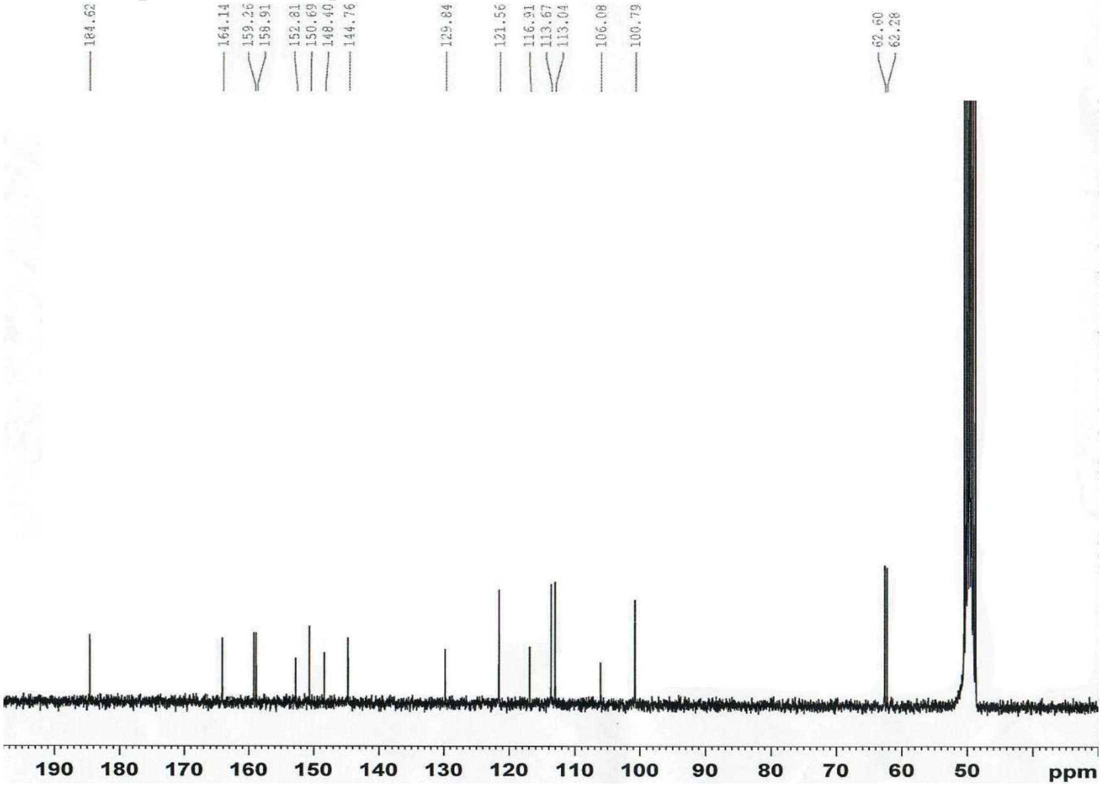

$^{1}$H NMR spectrum of compound **7** (DMSO-$d_6$, 300 MHz)

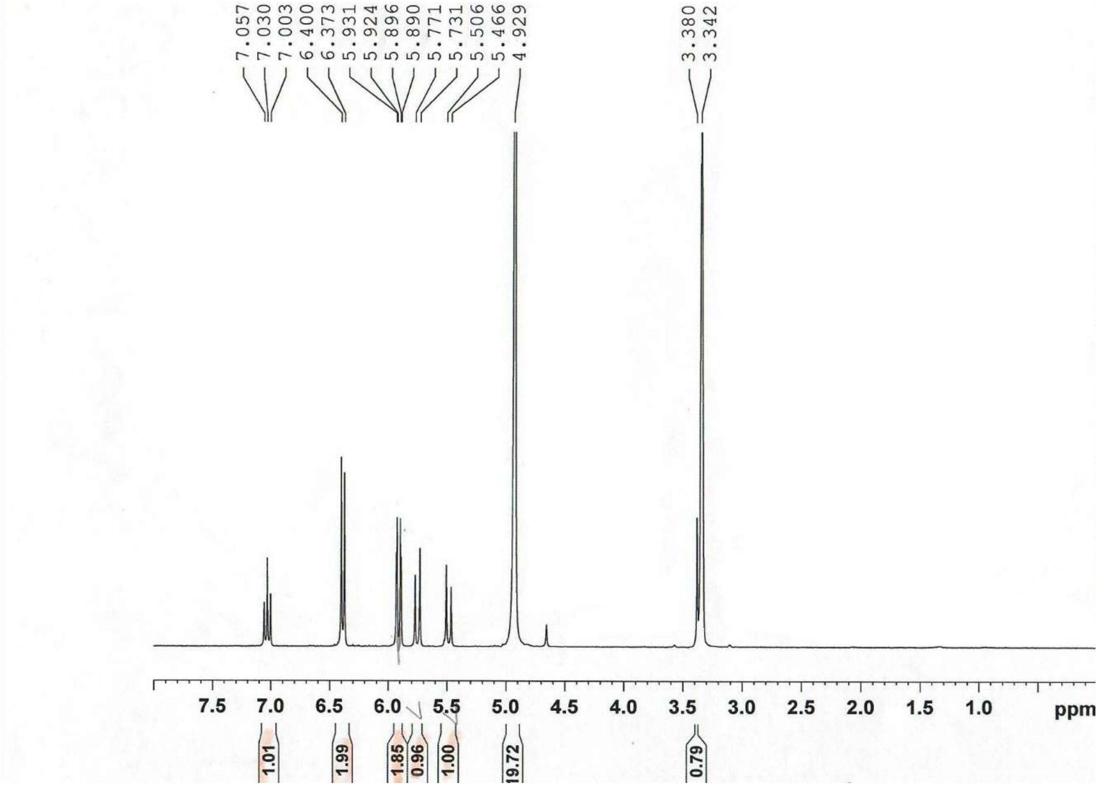


$^{13}$C NMR spectrum of compound **7** (DMSO-$d_6$, 75 MHz)

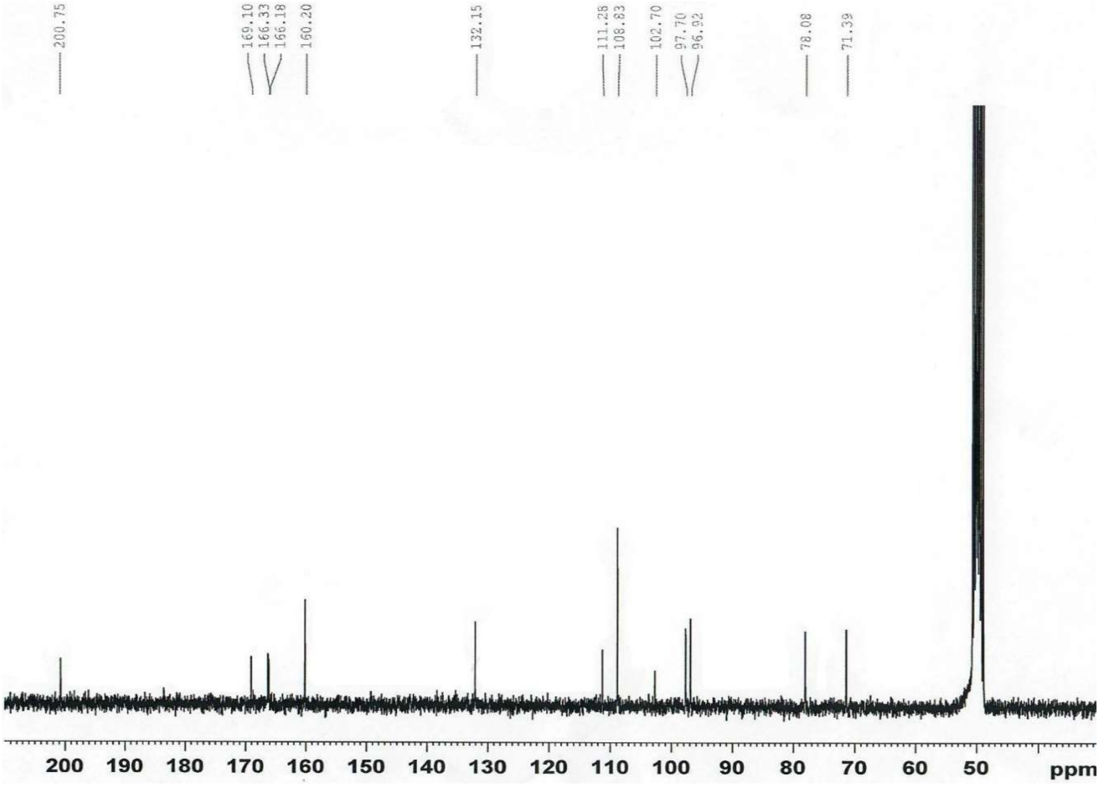

$^{1}$H NMR spectrum of compound **8** (DMSO-$d_6$, 600 MHz)

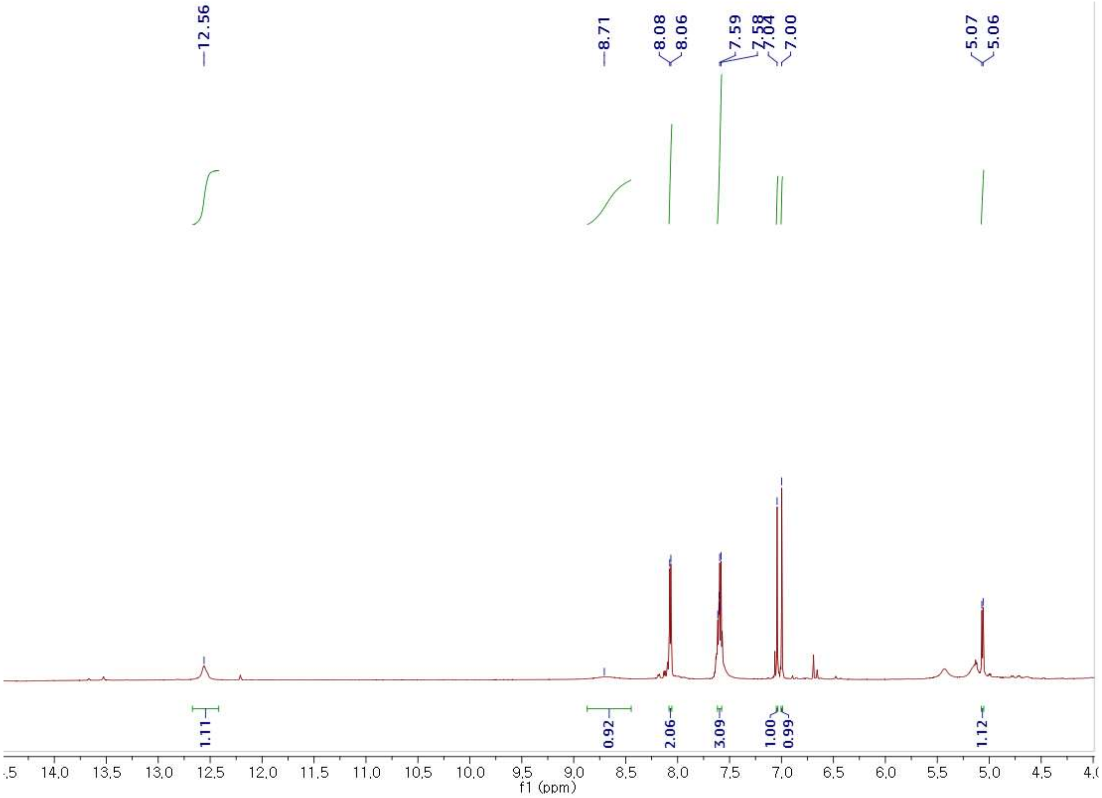


$^{13}$C NMR spectrum of compound **8** (DMSO-$d_6$, 125 MHz)

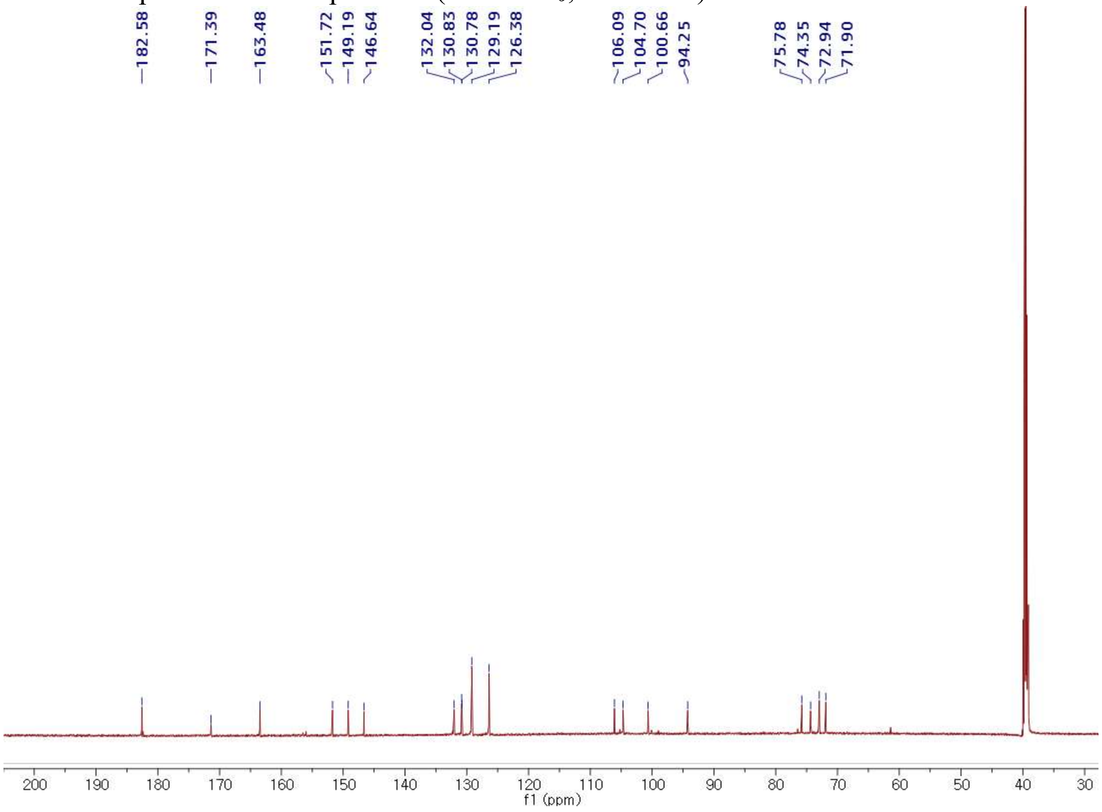

$^{1}$H NMR spectrum of compound **9** (DMSO-$d_6$, 600 MHz)

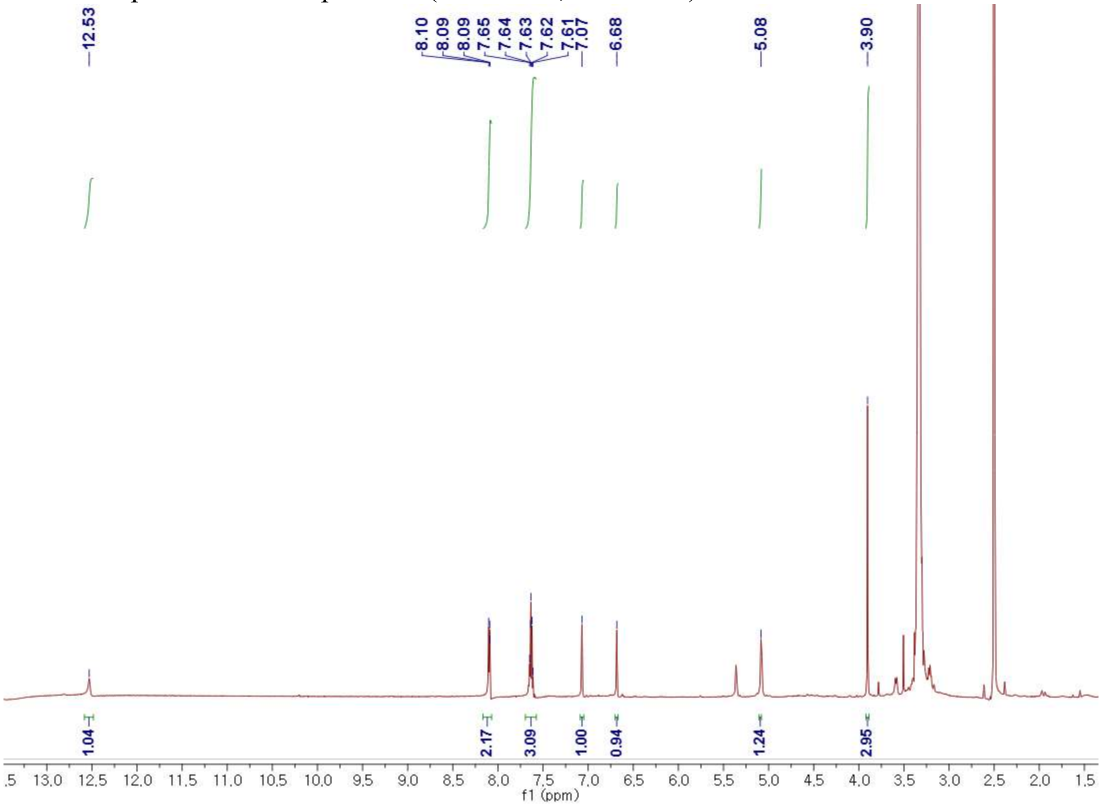


$^{13}$C NMR spectrum of compound **9** (DMSO-$d_6$, 125 MHz)

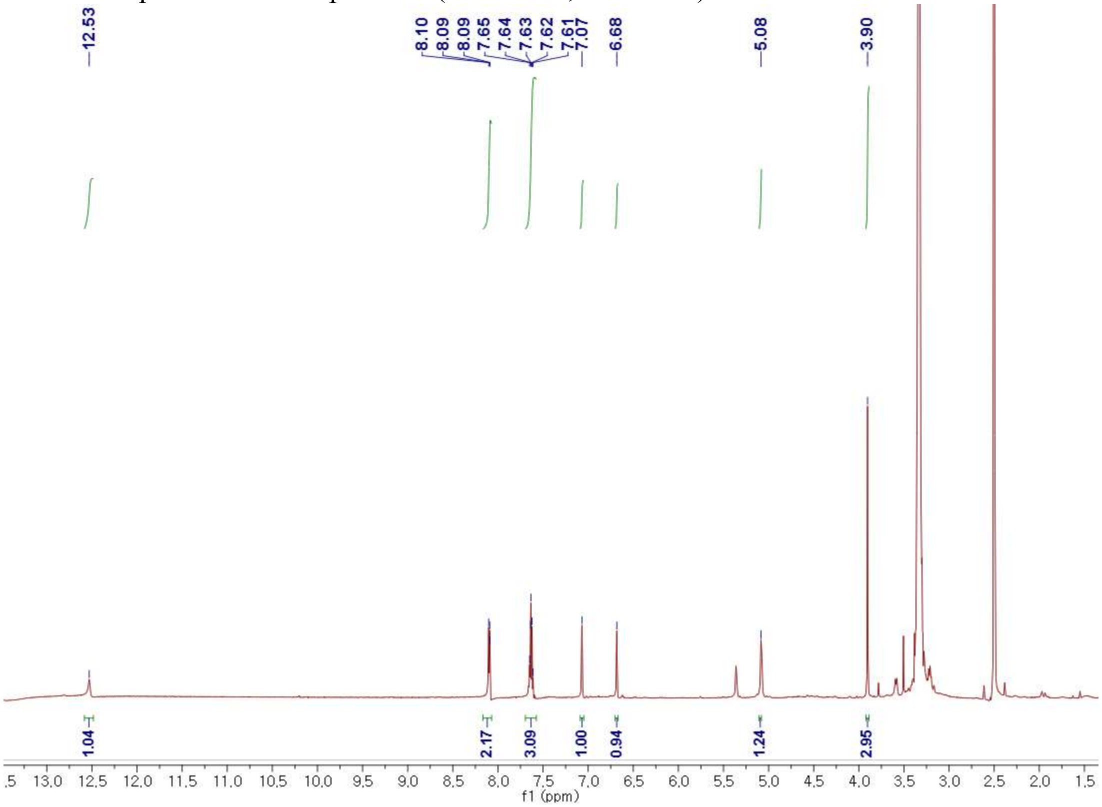

**Figure 3S** Sigmoidal dose-response curves of compounds on DPPH radical scavenging activity

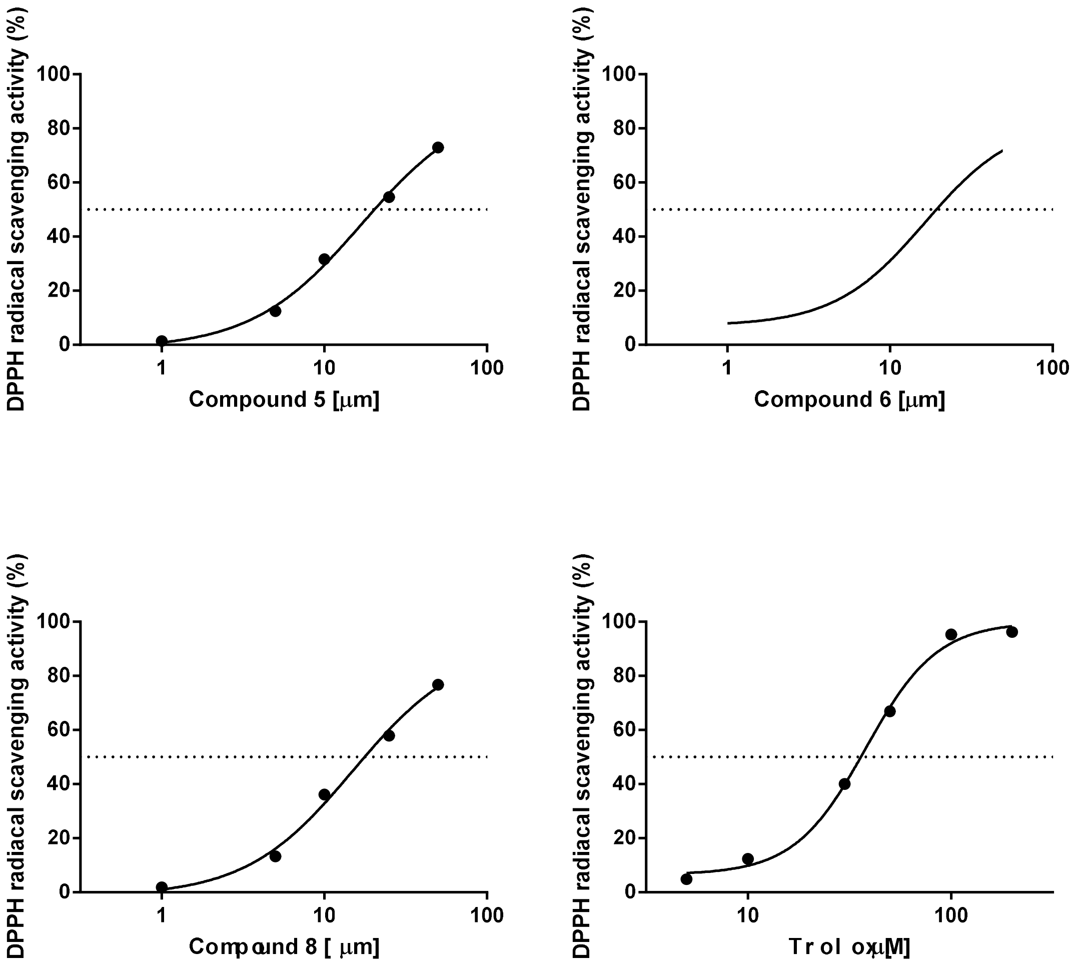

**Figure 4S** Sigmoidal dose-response curves of compounds on ABTS radical scavenging activity

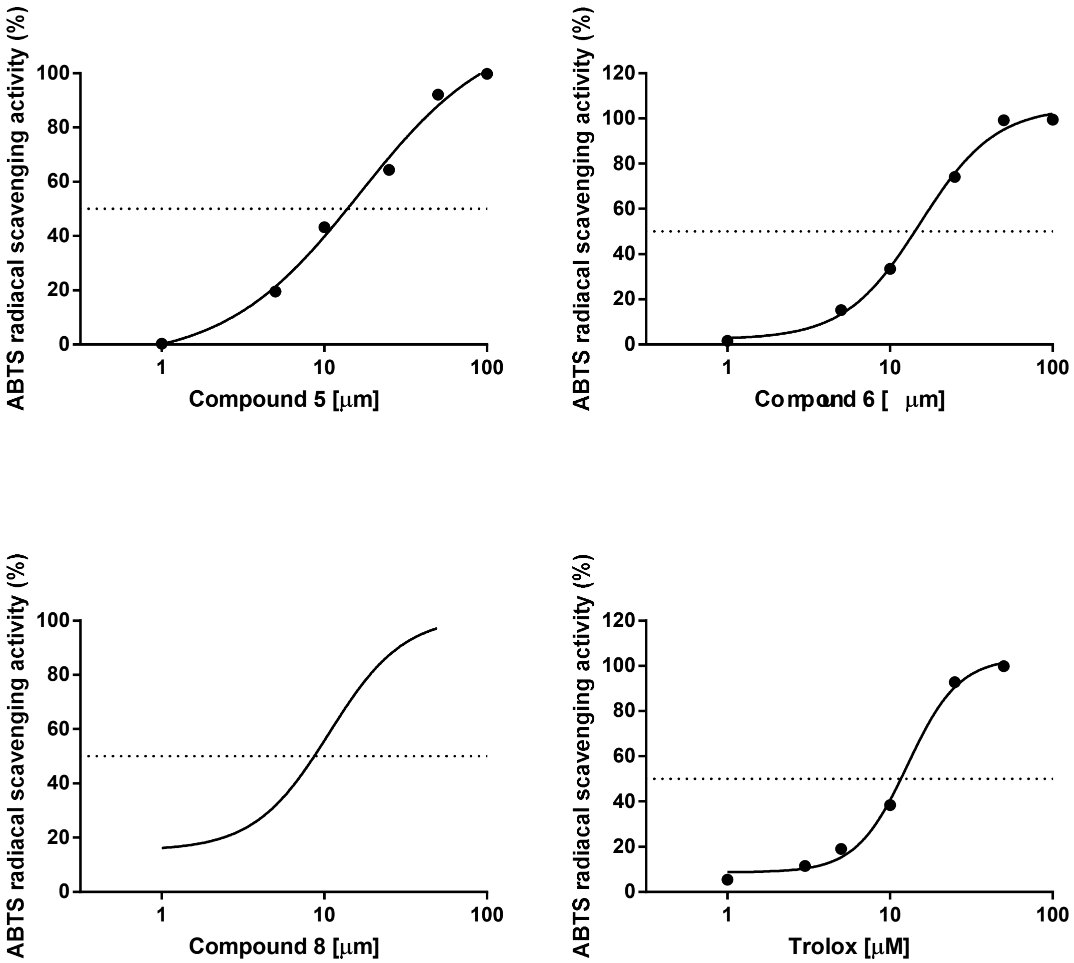

Supplement: Supplementary file 1 [file molecules-25-03617-s001.pdf]
